# Proximity-based defensive mutualism between *Streptomyces* and *Mesorhizobium* by sharing and sequestering iron

**DOI:** 10.1093/ismejo/wrad041

**Published:** 2024-01-10

**Authors:** Xueyuan Du, Ning Liu, Bingfa Yan, Yisong Li, Minghao Liu, Ying Huang

**Affiliations:** State Key Laboratory of Microbial Resources, Chinese Academy of Sciences, Institute of Microbiology, Beijing 100101, P. R. China; College of Life Sciences, University of Chinese Academy of Sciences , Beijing 101408, P. R. China; National Engineering Laboratory for Site Remediation Technologies, BCEG Environmental Remediation Co., Ltd., Beijing 100015, P. R. China; State Key Laboratory of Microbial Resources, Chinese Academy of Sciences, Institute of Microbiology, Beijing 100101, P. R. China; State Key Laboratory of Microbial Resources, Chinese Academy of Sciences, Institute of Microbiology, Beijing 100101, P. R. China; College of Life Sciences, University of Chinese Academy of Sciences , Beijing 101408, P. R. China; School of Public Health, Qingdao University, Qingdao 266071, P. R. China; State Key Laboratory of Microbial Resources, Chinese Academy of Sciences, Institute of Microbiology, Beijing 100101, P. R. China; State Key Laboratory of Microbial Resources, Chinese Academy of Sciences, Institute of Microbiology, Beijing 100101, P. R. China; College of Life Sciences, University of Chinese Academy of Sciences , Beijing 101408, P. R. China

**Keywords:** Streptomyces, Mesorhizobium, interaction, iron, siderophore, iron-porphyrin, proximity-based defensive mutualism

## Abstract

Microorganisms living in soil maintain intricate interactions among themselves, forming the soil microbiota that influences the rhizosphere microbiome and plant growth. However, the mechanisms underlying the soil microbial interactions remain unclear. *Streptomyces* and *Mesorhizobium* are commonly found in soil and serve as plant growth-promoting rhizobacteria (PGPR). Here, we identified an unprecedented interaction between the colonies of red-soil-derived *Streptomyces* sp. FXJ1.4098 and *Mesorhizobium* sp. BAC0120 and referred to it as “**p**roximity-**b**ased **d**efensive **m**utualism (PBDM).” We found that metabolite-mediated iron competition and sharing between the two microorganisms were responsible for PBDM. *Streptomyces* sp. FXJ1.4098 produced a highly diffusible siderophore, desferrioxamine, which made iron unavailable to co-cultured *Mesorhizobium* sp. BAC0120, thereby inhibiting its growth. *Streptomyces* sp. FXJ1.4098 also released poorly diffusible iron-porphyrin complexes, which could be utilized by *Mesorhizobium* sp. BAC0120, thereby restoring the growth of nearby *Mesorhizobium* sp. BAC0120. Furthermore, in ternary interactions, the PBDM strategy contributed to the protection of *Mesorhizobium* sp. BAC0120 close to *Streptomyces* sp. FXJ1.4098 from other microbial competitors, resulting in the coexistence of these two PGPR. A scale-up pairwise interaction screening suggested that the PBDM strategy may be common between *Mesorhizobium* and red-soil-derived *Streptomyces*. These results demonstrate the key role of iron in complex microbial interactions and provide novel insights into the coexistence of PGPR in soil.

## Introduction

The soil microbiome is populated by taxonomically diverse bacteria and fungi, giving rise to numerous intra- and inter-kingdom interactions that affect the rhizosphere microbiome and plant health [1–3]. Microorganisms that provide life support and/or aid in the defense of their host plants are called plant growth-promoting rhizobacteria (PGPR), which are involved in a variety of activities that promote plant growth, such as nutrient absorption, environmental stress tolerance, and pathogen inhibition [4, 5]. Streptomycetes, well-known producers of bioactive metabolites [6], are validated as PGPR that protect plants from pathogen infections and promote their growth [7–10]. In turn, the plants can reciprocally benefit the *Streptomyces* strains with nutrients through root exudates [11]. Rhizobia represent another major group of PGPR that can colonize inside the roots of legumes to form root nodules and fix nitrogen for their host plants [12–14]. Although streptomycetes and the rhizobia are from different phyla, *Actinomycetota* (earlier synonym: *Actinobacteria*) and *Pseudomonadota* (earlier synonym: *Proteobacteria*), respectively [15], they may synergistically promote plant growth. For example, co-inoculation of selected *Streptomyces* strains with *Mesorhizobium ciceri* has been shown to significantly enhance chickpea growth by promoting nodulation, increasing nitrogen fixation, and improving the host resistance against Botrytis gray mold disease [8]. However, the mechanism underlying this synergistic effect remains unclear.

In addition to their relationships with host plants, various interactions occur among the soil microorganisms. *Streptomyces* generally secrete secondary metabolites including antibiotics and siderophores into the environment, thereby exerting antagonistic effects on their neighbors [16–18]. We previously reported that the secondary metabolites produced by *Streptomyces* play a pivotal role in interference competition with other co-occurring soil microorganisms [19]. A recent study revealed that *Streptomyces* spores can hitchhike on the flagella of motile bacteria to be transported to plant roots, where the spores may germinate and produce antibiotics to ward off the plant pathogens [20]. Unlike *Streptomyces*, interactions of rhizobia with other microorganisms have long been underestimated. Zhang *et al*. reported that the mycelia of *Phomopsis liquidambaris* constitute ideal dispersal networks to facilitate rhizobial enrichment in the peanut rhizosphere from bulk soil, thereby triggering peanut–rhizobium nodulation [21]. However, interactions between *Streptomyces* and rhizobia and their effects on other organisms in the same habitat have rarely been studied. Moreover, antagonistic *Streptomyces* can indiscriminately kill other PGPR in the same niche, raising questions on how *Streptomyces* and rhizobia can coexist in the rhizosphere of legumes.

Microorganisms inhabiting the same niche often compete for limited resources. Although iron is one of the most abundant elements on Earth, it is normally present in its poorly soluble ferric ion (Fe[III]) form under aerobic environmental conditions, making it a limited resource in soil [22]. Thus, iron competition plays an important role in the soil microbial interactions, influencing the composition and function of soil microbiomes [23]. To effectively utilize poorly bioavailable iron, many soil microorganisms produce siderophores, a group of small-molecule chelators with high affinity for Fe(III) [24]. Siderophores play a well-established role in iron homeostasis within cells and exert two opposing social effects on microbial community members. Siderophores can be “public bads” [25], inhibiting the growth of microorganisms lacking matching receptors required for iron uptake by sequestering environmental iron. Many PGPR, including *Streptomyces*, suppress phytopathogen growth by producing siderophores [7, 23, 24]. In contrast, siderophores can also be “public goods” [25], facilitating the growth of microorganisms with matching receptors by delivering iron to the cells. For example, desferrioxamine E (DFOE) produced by *Streptomyces coelicolor* can be readily pirated by the DFO-deficient *Amycolatopsis* sp. AA4 [26], and *Sinorhizobium meliloti* can acquire iron from xenosiderophores, ferrichrome, and ferrioxamine B [27].

In addition to siderophores, heme and heme-containing proteins are another major source of iron for microorganisms. The ability to use heme or heme complexes as iron source was previously believed to be restricted to animal-pathogenic bacteria until Noya *et al*. discovered this ability in some non-pathogenic bacterial genera in 1997 [28–30]. Many bacteria have developed specialized receptors and uptake systems to acquire heme- or iron-containing porphyrins from their hosts or surroundings [31, 32]. In Rhizobia, such as *Bradyrhizobium japonicum* [29], *Rhizobium leguminosarum* [33], and *S. meliloti* [34], heme can be recognized by specific TonB-dependent outer membrane receptors and subsequently transported into the cell via ATP binding cassette (ABC) transporters with the energy provided by the TonB–ExbB–ExbD complex [35]. However, the combined effects of siderophores and heme uptake strategies for iron competition on the interactions among soil microorganisms remain obscure.

Red soil (equivalent to Ultisol in the US soil classification system or Acrisol and Ferralsol in the soil classification of the Food and Agriculture Organization of the United Nations) is characterized by high iron oxide content, low organic matter, and acidity, and is widespread in tropical and subtropical regions [36]. Previous studies have demonstrated that red soil contains abundant *Streptomyces* and rhizobia [19, 37]. In this study, unlike classical microbial interactions represented by growth inhibition and facilitation, we discovered an unprecedented interaction that some red-soil-derived streptomycetes inhibited the growth of *Mesorhizobium* spp. at a certain distance, while not affecting those in the vicinity. We termed this phenomenon “**p**roximity-**b**ased **d**efensive **m**utualism (PBDM)” between *Streptomyces* and *Mesorhizobium* and demonstrated that this interaction is mediated by a combination of iron sequestration and sharing, rather than physical contact. DFOE and iron-porphyrin complexes produced by *Streptomyces* are responsible for the sequestration and sharing of iron, respectively, resulting in simultaneous antagonism and amity between different bacterial phyla. In addition, to evaluate the ecological significance of the interaction, we carried out ternary interactions in which a bacterium co-isolated from the same red-soil sample or a common plant pathogen was added to the *Streptomyces* versus *Mesorhizobium* co-culture. The results showed that *Streptomyces* could partially protect *Mesorhizobium* from microbial competitors. Our findings provide new insights into the coexistence of PGPR of different microbial phyla in soil and highlight the importance of iron in microbial interactions.

## Materials and methods

### Microbial strains and cell suspensions

All strains and media used in this study are listed in Tables S1 and S2, respectively. *Streptomyces* and co-isolated bacteria were cultured in 50 ml liquid GYM medium [19, 37], and rhizobial strains were cultured in 50 ml liquid TY medium [38] for 72 h at 28°C and 200 rpm. Fungi were cultured in 50 ml liquid potato dextrose broth, and *Agrobacterium rubi* CGMCC 1.2555^T^ was cultured in 50 ml liquid Luria–Bertani medium for 48 h at 28°C and 200 rpm. Cells were harvested at the exponential growth phase, and the culture medium was washed off with double-distilled water. Then, the cells were resuspended in 50 ml double-distilled water for subsequent interaction experiments. For additional iron treatment, 100 mM FeCl_2_ and FeCl_3_ stocks were diluted to the required concentrations and added to the bacterial suspensions.

### Interaction experiments and classification of different interaction categories

**Figure 1:**
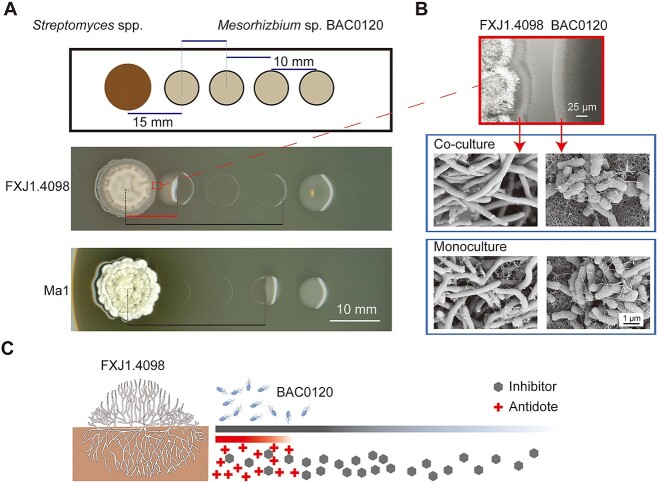
PBDM interaction between *Streptomyces* and *Mesorhizobium* and the putative mechanism; (A) PBDM and competitive interactions between *Streptomyces* and *Mesorhizobium*; *Mesorhizobium* strains were inoculated onto the culture plates 4 days after the inoculation of *Streptomyces* strains; pictures were taken after 4 days of co-culture and a representative picture is shown for each pair; *Streptomyces* sp. FXJ1.4098 vs. *Mesorhizobium* sp. BAC0120 pair showed the unprecedented PBDM phenomenon (*n* = 43), and *Streptomyces albidoflavus* Ma1 vs. *Mesorhizobium* sp. BAC0120 pair showed a common competition phenomenon (*n* = 3); horizontal black and red solid lines indicate the radii of inhibition and recovery zones of *Mesorhizobium* by *Streptomyces*, respectively; (B) representative microscopy images of the edge areas in the co-culture and monocultures of *Streptomyces* sp. FXJ1.4098 and *Mesorhizobium* sp. BAC0120; no physical contact was detected between the two strains in the co-culture (*n* = 3); scanning electron micrographs of the monocultures were taken after the incubation of *Streptomyces* sp. FXJ1.4098 for 8 days and *Mesorhizobium* sp. BAC0120 for 4 days (*n* = 3); (C) schematic diagram of putative metabolite-mediated PBDM interaction between *Streptomyces* sp. FXJ1.4098 and *Mesorhizobium* sp. BAC0120; black and red gradient lines indicate different diffusion distances of the inhibitor and antidote, respectively.

Pairwise interactions between *Streptomyces* and rhizobia were carried out on Petri dishes containing GYM agar. *Streptomyces* was inoculated 4 days ahead of rhizobia to ensure the directionality of the interactions. *Streptomyces* strains were inoculated by drop plating 10 μl of the aforementioned storage suspensions onto the agar and cultured at 28°C for 4 days to form a colony ~10 mm in diameter. Then, 10-μl suspensions of rhizobial strains were drop plated 15, 25, 35, and 45 mm to the right from the center of the *Streptomyces* colony in a straight line (Fig. 1A). Growth of both *Streptomyces* and rhizobial strains was recorded for the next 4 days of co-culture. Competition was determined if a clearing zone of rhizobia was observed in the area adjacent to the *Streptomyces* colony; PBDM was determined if the rhizobia grew well near the *Streptomyces* but were inhibited at a greater distance, i.e., the growth of rhizobia was partially recovered near the *Streptomyces* but remained inhibited at a certain distance; and neutrality was determined if the growth of *Streptomyces* and rhizobia was consistent with that when cultured alone.

Pairwise interaction experiments between co-isolated bacteria, or plant pathogens and *Mesorhizobium* sp. BAC0120, *Streptomyces* sp. FXJ1.4098 and FXJ1.4098Δ*desD* [19] were conducted as follows: volumes of 10-μl suspensions of individual microorganisms were simultaneously spotted onto the GYM plate at a distance of 1.5 cm (between the centers of the two droplets). Interaction results were recorded after 4 days of co-culture at 28°C.

#### Ternary interaction experiment

About 10 μl of *Streptomyces* sp. FXJ1.4098 suspensions were drop plated onto a GYM plate and cultured for 4 days to form a colony. Then, 10-μl suspensions of *Mesorhizobium* sp. BAC0120 and a co-isolated bacterium or a plant pathogen were individually and simultaneously spotted onto the plate 1.5 cm away from each other. Interaction results were recorded after 4 days of co-culture at 28°C.

### Scanning electron microscopy

Samples of colonies scraped from plates were fixed in 2.5% paraformaldehyde–glutaraldehyde mixture buffered with 0.1 M phosphate (pH 7.2) for 2 h and postfixed in 1% osmium tetroxide with the same buffer for 1 h. After fixation, the samples were dehydrated in a gradient of ethanol and substituted with isoamyl acetate. Finally, the samples were dried to the critical point with CO_2_ and sputtered with gold in a sputter coater (Leica EM CPD300, Germany) before being subjected to scanning electron microscopy (Hitachi SU8010, Japan).

### Metabolite extraction and chemical analysis

Pairwise interactions between *Streptomyces* and *Mesorhizobium* were conducted as described above using 40 Petri dishes. After 4 days of co-culture, the plates were divided into four areas based on the colony growth. Agar from each area (~42, 54, 372, and 532 ml of agar was collected from areas a to d, respectively) was separately extracted thrice with equal volumes of ethanol and thrice with equal volumes of ethyl acetate for compounds with different hydrophilicities. Organic phases were concentrated to dryness under vacuum and the residues were redissolved in 2 ml of methanol. Extracts of areas a–d were then normalized and diluted according to their original volumes before high-performance liquid chromatography (HPLC) analysis. Ultra HPLC and high-resolution mass spectrometry (UHPLC-HRMS) analyses were conducted as previously described [39], with slight modifications. Briefly, 20 μl of each dilution was analyzed on a Shimadzu Prominence HPLC system using a Waters Xbridge octadecyl silane column with a linear gradient of MeOH–H_2_O (each containing 0.1% formic acid) from 20:80 to 100:0 over 15 min at a flow rate of 1 ml/min. The effluent was monitored at 200–800 nm.

To detect the production of siderophores, chrome azurol S (CAS) assay was performed as previously described [40], with a slight modification that the co-culture plates were overlaid with CAS agar.

### Genome sequencing

All plasmids used in this study are listed in Table S3. Genomic DNAs of *Streptomyces* and *Mesorhizobium* were extracted following the Kirby mix procedure [41]. Genomic DNA of *Streptomyces* sp. FXJ1.4098 was sequenced using PacBio RS II at the Beijing Institute of Genomics (Chinese Academy of Sciences). The PacBio SMRT-Analysis package (https://www.pacb.com) with default parameters was used for the quality control of the raw polymerase reads. *De novo* assembly was conducted with the high-quality SMRT long reads by Canu software [42]. A total of 12 780 912 bp of genome sequence distributed across 10 contigs was obtained.

Genomic DNA of *Mesorhizobium* sp. BAC0120 was sequenced using a NovaSeq 6000 platform (Illumina) in 150-bp paired-end mode at Novogene Bioinformatics Technology Co., Ltd, Beijing, China. All good-quality paired reads were assembled using SOAP denovo [43] and a total of 7 198 477 bp genome sequence distributed across 50 contigs was obtained.

### Genetic manipulation

Disruptions of the genes encoding the TonB-dependent receptor (TBDR gene), ATPase component (*hmuV*), and permease protein (*hmuU*) of the ABC-type hemin transport system in *Mesorhizobium* sp. BAC0120 were performed using suicide plasmid pJQ200SK-based insertional inactivation via single homologous recombination. Taking *hmuV* as an example, primers BAC-*hmuV*-F and BAC-*hmuV*-R (Table S4) were used to amplify a 464-bp internal fragment of the *hmuV* coding region using *Mesorhizobium* sp. BAC0120 genomic DNA as the template. The fragment was ligated to the PstI-digested plasmid pJQ200SK to generate pJQ::*hmuV* using Gibson assembly [44]. The recombinant plasmid was introduced into *Mesorhizobium* sp. BAC0120 via conjugation with the help of *Escherichia coli* Top 10 (Invitrogen, USA) carrying the helper plasmid, pRK2013 [45]. Recombination of the cloned *hmuV* fragment in the suicide plasmid with its homologous counterpart in the *Mesorhizobium* sp. BAC0120 chromosome resulted in the disruption of *hmuV* gene. Gentamicin (30 μg/ml) and trimethoprim (10 μg/ml) were used for the genetic manipulation and screening of mutant strains. Transconjugants sensitive to gentamicin were selected as the pJQ200SK-cured mutants. The resultant *Mesorhizobium* mutants were confirmed via polymerase chain reaction (PCR) and subsequent sequencing. Primer sequences used in the validation experiment are listed in Table S4.

### Transcriptional analysis

Total RNAs were isolated from monocultured or co-cultured *Streptomyces* sp. FXJ1.4098 and *Mesorhizobium* sp. BAC0120 grown on GYM agar at corresponding time points using the QIAGEN Rneasy mini kit, and further purified using RQ1 Rnase-free Dnase I (Promega, USA), according to the manufacturer’s instructions. RNA quality was assessed using a NanoDrop spectrophotometer (ND-1000). Approximately 750 ng of purified RNA was reverse transcribed to generate cDNA using the SuperRT cDNA Synthesis Kit (CWBIO, China). RNA-sequencing (RNA-seq) was performed by Novogene Bioinformatics Technology Co., Ltd on the NovaSeq 6000 platform (Illumina), and paired-end reads of 150-bp length were generated.

Reverse transcription-quantitative polymerase chain reaction (RT-qPCR) was performed using the FastFire qPCR PreMix kit (SYBR Green) and a LightCycler 480 II, according to the manufacturers’ instructions. Moreover, the 16S rRNA gene was used as an internal control with the qBAC-16S-F/qBAC-16S-R primer pair. Three independent biological replicates were analyzed in all RT-qPCR experiments. The data represent the average of three replicates. All primer sequences are listed in Table S4.

### Gene set enrichment analysis

Differentially expressed pathways were identified by comparing the transcriptome data from monocultures and co-cultures. A gene expression matrix was constructed according to the format of the example datasets on the official website of Broad Institute (http://software.broadinstitute.org/gsea/datasets.jsp). Monocultured *Streptomyces* sp. FXJ1.4098 was used as the control group for comparison with co-cultured *Streptomyces* sp. FXJ1.4098, and the sample grouping information was organized. Gene matrix-transposed file of *Streptomyces* sp. FXJ1.4098 was generated using the clusterProfiler package based on the RAST (https://rast.nmpdr.org) [46] and Gene Ontology/Kyoto Encyclopedia of Genes and Genomes annotation files of *Streptomyces* sp. FXJ1.4098. Gene set enriched in co-cultured *Streptomyces* sp. FXJ1.4098 compared to its monoculture was analyzed using Gene Set Enrichment Analysis (GSEA) software [47]. Normalized enrichment score (|NES| > 1), false-discovery rate <0.25, and *P*-value <.05 were used to quantify the enrichment magnitude and statistical significance.

### Phylogenetic analysis

The 16S rRNA genes of *Streptomyces* spp. FXJ1.4033 (GenBank accession number MT826305), FXJ1.4034 (MT826306), and FXJ1.4038 (MT826310) and *Mesorhizobium* spp. BAC0120 (MT826559) and BAC0074 (MT826517) were amplified and sequenced using universal primers 27f and 1492r [48]. The 16S rRNA genes of the other *Streptomyces* and *Mesorhizobium* strains used in this study (Table S1) were obtained from their genomic sequences retrieved from the GenBank database. The phylogenetic trees based on the 16S rRNA gene sequences were constructed with MEGA 11 [49] using the Maximum Likelihood method [50]. The evolutionary distance was computed using Kimura’s two-parameter method [51] with 1000 bootstrap replicates.

### Statistical analysis

Unless otherwise stated, all experiments were performed independently in triplicate. The figures of interactions and chemical analyses in this paper show a representative experiment. The radii of inhibition and recovery zones of rhizobial growth were measured using PixelStick (version 2.16.2; PlumAmazing Softwares, Princeville, HI). The radius of inhibition zone was obtained by measuring the radial distance from the center of the *Streptomyces* colony (the drop-plating point) to the left edge of the rhizobial colony that grew on the right of the inhibition area (Fig. 1A). The radius of recovery zone was obtained by measuring the radial distance from the center of the *Streptomyces* colony to the right edge of the recovered rhizobial colony on the left of the inhibition area (Fig. 1A). Data are represented as the mean ± standard deviation (*n* = 3) or median (*n* = 40). Individual sample size and statistical test used in each experiment are indicated in the figure legends. Statistical significance was analyzed using an unpaired two-tailed Student’s *t-*test. A *P*-value <.05 was considered statistically significant.

## Results

### Unprecedented PBDM interaction between *Streptomyces* and *Mesorhizobium*

In our previous work, we tested thousands of pairwise interactions between red-soil-derived actinobacteria and other co-occurring microorganisms and found that neutral effects dominated these interactions [19]. In addition to the documented interaction types of competition, facilitation, and neutrality, we observed a puzzling phenomenon between *Streptomyces* sp. FXJ1.4098 and *Mesorhizobium* sp. BAC0120 isolated from the same sample. Unlike the commonly observed growth inhibition of *Mesorhizobium* sp. BAC0120 by other streptomycetes, *Mesorhizobium* sp. BAC0120 colonies close to and far away from *Streptomyces* sp. FXJ1.4098 grew well, while those located at a moderate distance were inhibited, resulting in a PBDM phenomenon (Fig. 1A).

Light and scanning electron microscopy revealed that neighboring *Streptomyces* sp. FXJ1.4098 and *Mesorhizobium* sp. BAC0120 colonies were separated from each other, excluding the possibility of direct physical contact during the PBDM interaction (Fig. 1B). Furthermore, no PBDM-like phenomena were observed when growing *Mesorhizobium* sp. BAC0120 on a plate after the removal of pre-cultured *Streptomyces* sp. FXJ1.4098, implying the essential role of the temporal and spatial co-occurrence of these two strains in inducing PBDM (Fig. S1). Therefore, we speculated that the PBDM phenomenon may be caused by the extracellular molecule-mediated interactions and proposed a plausible mode of interaction: *Streptomyces* sp. FXJ1.4098 secretes at least two disparate metabolites, an inhibitor with good diffusivity and an antidote with poor diffusivity (Fig. 1C).

### *Streptomyces* sp. FXJ1.4098 inhibits the growth of *Mesorhizobium* sp. BAC0120 at a distance by producing DFOE that sequesters iron

To verify the metabolite-mediated PBDM interaction proposed above, we analyzed the metabolites extracted from different areas of the FXJ1.4098 versus BAC0120 co-culture plate. The plate was divided into four annular areas (**a**, **b**, **c**, and **d**) based on the colony growth (2A). After removing the colonies, the agar of each area was extracted, and the extracts were subjected to bioactivity assays. Unlike the extract from area **d**, those from areas **a** to **c** exhibited excellent inhibition against *Mesorhizobium* sp. BAC0120 (2B and Fig. S2). HPLC profiling detected a metabolite present in areas **a**–**c**, but absent in area **d** (2C and Fig. S3). The metabolite was identified as DFOE, a siderophore commonly found in *Streptomyces*, based on analysis of its UHPLC-HRMS, ultraviolet (UV) spectrum, and MS/MS data (Fig. S4). Previous studies have shown that *Streptomyces* can produce a series of different DFO analogs [52], but *Streptomyces* sp. FXJ1.4098 produces only DFOE (Fig. S5).

**Figure 2:**
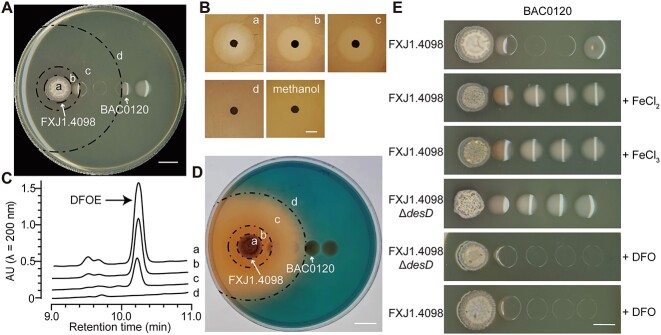
*Streptomyces* sp. FXJ1.4098 inhibits the growth of *Mesorhizobium* sp. BAC0120 via iron sequestration mediated by desferrioxamine (DFO) siderophores; (A) division of the interaction plate into four areas; areas are divided by dotted arcs; (a) area where *Streptomyces* sp. FXJ1.4098 grew; (b) area where *Mesorhizobium* sp. BAC0120 recovered from inhibition; (c) area where *Mesorhizobium* sp. BAC0120 was inhibited; (d) control area where the growth of *Mesorhizobium* sp. BAC0120 was not affected by *Streptomyces* sp. FXJ1.4098; (B) bioactivity assay of extracts from areas a–d of co-culture plates against *Mesorhizobium* sp. BAC0120 (*n* = 3); after normalization, 100 μl of fermentation extracts from areas a–d was used for bioactivity assay; “Methanol” indicates the control of 100 μl solvent; quantification of the result is shown in Fig. S2; (C) a representative HPLC profile showing a peak of DFOE exclusively shared among the extracts from areas a–c in the co-culture plate; corresponding intact HPLC profile is shown in Fig. S3, and the chemical elucidation of DFOE is shown in Fig. S4; (D) detection of DFOE, a siderophore, distribution in the co-culture plate using an overlay of CAS agar; the orange area indicates the chelation of iron by DFOE; dotted arcs indicate the same divided areas shown in (A); (E) effects of DFO and iron ions on the interaction between *Streptomyces* sp. FXJ1.4098 and *Mesorhizobium* sp. BAC0120; pairwise experiment was performed as described in Fig. 1; FXJ1.4098Δ*desD*, *Streptomyces* sp. FXJ1.4098 mutant in which the core biosynthetic gene *desD* for producing DFOs was disrupted; +FeCl_2_ and +FeCl_3_, *Mesorhizobium* sp. BAC0120 suspensions with 1 mM FeCl_2_ and FeCl_3_, respectively; +DFO, medium with 200 μM DFO; scale, 10 mm.

We used the CAS assay to examine the distribution of DFOE on the PBDM plate (2D). Consistent with HPLC profiles above (2C), DFOE exhibited a gradient distribution from areas **a** to **c** (where CAS turned orange), while its concentration in area **d** fell below the detection limit of the CAS assay (where CAS remained blue) (2D). A commercially available analog of DFOE, deferoxamine mesylate salt (DFOB) could also simulate the iron-sequestrating effect of DFOE during this interaction, confirming that DFO inhibits the growth of *Mesorhizobium* sp. BAC0120 (Fig. S6). Moreover, the DFO concentration (*c*_DFO_) was found to be positively correlated with the radius of inhibition zone (ri) against *Mesorhizobium* sp. BAC0120 (Fig. S7A), conforming to an exponential fitting function (*c*_DFO_ = 0.04352e^0.1986ri^, *R*^2^ = .99; Fig. S7B). In the co-culture plates, the radius of inhibition zone caused by *Streptomyces* sp. FXJ1.4098 ranged from 35.7 to 43.1 mm with a median of 38.1 mm (*n* = 40; Fig. S8A).

To determine whether growth inhibition of *Mesorhizobium* sp. BAC0120 was caused by DFO-mediated iron deficiency, 1 mM FeCl_2_ or FeCl_3_ was added to the *Mesorhizobium* sp. BAC0120 cell suspension prior to inoculation for pairwise interactions. Indeed, all *Mesorhizobium* sp. BAC0120 colonies containing exogenous iron ions were not inhibited by *Streptomyces* sp. FXJ1.4098 (2E).

To further verify the inhibitory effect of DFOE *in vivo*, we performed a pairwise interaction experiment using the DFO-deficient strain, FXJ1.4098Δ*desD* [19] and found that it no longer inhibited *Mesorhizobium* sp. BAC0120 (2E). In addition, the PBDM phenomenon reappeared when FXJ1.4098Δ*desD* and *Mesorhizobium* sp. BAC0120 were co-cultured in a plate containing 200 μM exogenous DFO (2E). These findings demonstrate that *Streptomyces* sp. FXJ1.4098 inhibits the growth of *Mesorhizobium* sp. BAC0120 via iron sequestration mediated by DFO secretion.

### Growth of *Mesorhizobium* sp. BAC0120 adjacent to *Streptomyces* sp. FXJ1.4098 is recovered by iron-porphyrin complexes secreted from *Streptomyces* sp. FXJ1.4098

**Figure 3:**
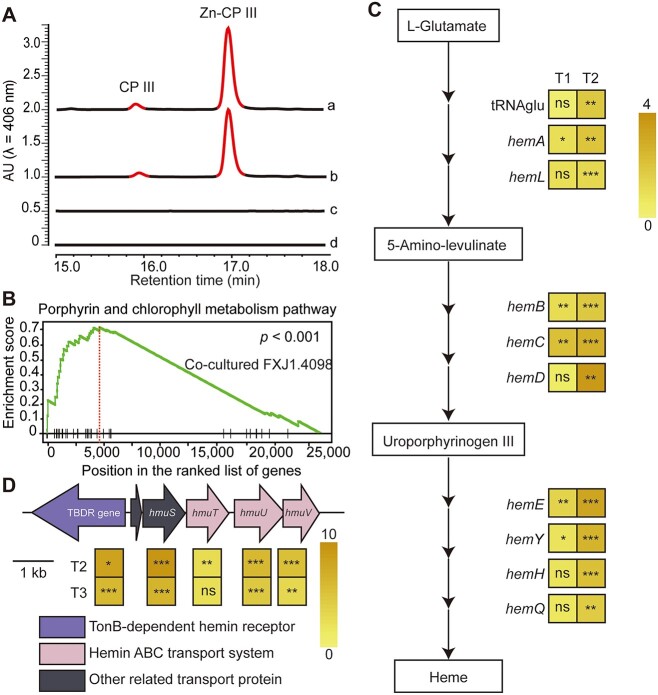
Iron-porphyrin is the putative antidote for the PBDM interaction between *Streptomyces* sp. FXJ1.4098 and *Mesorhizobium* sp. BAC0120; (A) representative HPLC profile showing detection of CPs in the extracts from areas a–d of co-culture plates of FXJ1.4098 vs. BAC0120 (*n* = 3); CP III and zincphyrin (Zn-CP III) were detected in the extracts from areas a and b; for complete HPLC profile, please refer to Fig. S9; (B) GSEA of the RNA-seq data; GSEA revealed significant enrichments of genes involved in porphyrin and chlorophyll metabolism in the transcriptome of co-cultured *Streptomyces* sp. FXJ1.4098 compared to that of monocultured *Streptomyces* sp. FXJ1.4098 at T1 (T1 = 6 d; see Fig. S13 for more information on time points); (C) the heme biosynthetic pathway and transcriptional analyses of heme biosynthetic genes in *Streptomyces* sp. FXJ1.4098 co-cultured with *Mesorhizobium* sp. BAC0120 via RT-qPCR; total RNAs were isolated from monocultured and co-cultured *Streptomyces* sp. FXJ1.4098 on days 6 (T1) and 7 (T2), respectively; (D) organization and transcriptional analyses of genes in the heme uptake system in *Mesorhizobium* sp. BAC0120 via RT-qPCR; total RNAs were isolated from monocultured and co-cultured *Mesorhizobium* sp. BAC0120 on days 3 (T2) and 4 (T3), respectively. Total RNAs were reverse transcribed to obtain cDNAs; the 16S rRNA gene was used as an internal reference to normalize the RNA concentration; yellow to brown square boxes indicate the upregulation of gene expression in the co-culture compared to that in the monoculture; color gradient corresponds to the log2 |fold-change| value. *P < .05; **P < .01; ***P < .001; ns, not significant.

Although the concentration of *Streptomyces* sp. FXJ1.4098-secreted DFOE was higher in co-culture plate area **b** than in area **c**, the growth of *Mesorhizobium* sp. BAC0120 was not inhibited in area **b** (2A and D). This paradox led to the hypothesis that area **b** may contain an unidentified antidote as an alternative iron resource for *Mesorhizobium* sp. BAC0120. The radius of recovery zone by *Streptomyces* sp. FXJ1.4098 ranged from 13.1 to 15.7 mm with a median of 14.3 mm (*n* = 40; Fig. S8B). HPLC analysis detected two products, F1 and F2, at 406 nm in both areas **a** and **b** of the co-culture plate, whereas almost no such products were detected in areas **c** and **d** of the same plate (Fig. 3A and Fig. S9).

Based on UHPLC-HRMS analysis, the accurate mass of F1 was found to be identical to that of coproporphyrin (CP) I, II, III, and IV and isocoproporphyrin, all of which share the same accurate mass of 654.2690 (*m/z* 655.2755 Da, [M + H]^+^). Because CP III is the most common CP produced in bacteria, we further compared the chemical data of the CP III standard with that of F1. Similar retention time and UV spectra in HPLC as well as MS/MS spectra in HRMS strongly indicated that F1 is CP III (Figs S10 and S11). The measured [M + H]^+^ of F2 (*m/z* 716.1807 Da) matched well with the [M-2H + Zn]^+^ of F1 (*m/z* 716.1812 Da), and the UV spectrum and distribution pattern of the isotopic peaks of F2 were consistent with those of the reported zinc complex, zincphyrin (Fig. S10) [53, 54]. Hence, it was deduced that F2 is the Zn^2+^ chelate of F1. In summary, products F1 and F2 were determined to be CP III and Zn-CP III with the molecular formulas C_36_H_38_N_4_O_8_ and C_36_H_36_N_4_O_8_Zn, respectively. However, since neither CP III nor Zn-CP III contains iron, neither CP III and Zn-CP III individually nor their combination restored the growth of *Mesorhizobium* sp. BAC0120 on a plate containing 200 μM exogenous DFO (Fig. S12).

To further trace the hints for the putative antidote, we then conducted transcriptomic analysis. Total RNAs from monocultured and co-cultured *Streptomyces* sp. FXJ1.4098 cells were extracted at time points T1 (6 or 4 + 2 d) and T2 (7 or 4 + 3 d) (Fig. S13) and subjected to RNA-seq analysis. GSEA revealed that five gene sets were significantly enriched at T1 in co-cultured *Streptomyces* sp. FXJ1.4098 compared to that in its monoculture (Table S5), but no gene sets were significantly enriched at T2. Among the five gene sets, we focused on those associated with iron metabolism and transport and found one set related to porphyrin and chlorophyll metabolism (*P* < .001) (Fig. 3B). Although the gene set for porphyrin and chlorophyll metabolism was not significantly upregulated at T2, most of the genes involved in heme (iron protoporphyrin IX) biosynthesis were upregulated at both T1 and T2 (Table S6). Therefore, we used RT-qPCR to determine the expression levels of genes involved in heme biosynthesis in *Streptomyces* sp. FXJ1.4098. Compared with those in the monoculture, the transcriptional levels of all genes involved in heme biosynthesis were upregulated in the co-culture at T2, and half of the genes were upregulated at T1 (Fig. 3C and Fig. S14). These results combined with the phenomenon shown in Fig. S1 suggest that the presence of *Mesorhizobium* sp. BAC0120 induces the upregulation of heme biosynthesis in *Streptomyces* sp. FXJ1.4098 during PBDM interaction.

Considering the biomass of *Mesorhizobium* sp. BAC0120 and the delay between gene expression and corresponding product formation in *Streptomyces* sp. FXJ1.4098, we examined the transcriptional levels of porphyrin uptake genes in *Mesorhizobium* sp. BAC0120 at T2 and T3 time points (T2 = 3 d and T3 = 4 d; Fig. S13). Coincidently, the TBDR gene (encoding TonB-dependent hemin receptor), *hmuS* (encoding hemin transport protein), *hmuT* (encoding periplasmic hemin-binding protein), *hmuU* (encoding permease protein of hemin ABC transporter), and *hmuV* (encoding ATPase component of the ABC-type hemin transport system) in co-cultured *Mesorhizobium* sp. BAC0120 were significantly upregulated compared to those in its monoculture at T2 and T3 (Fig. 3D and Fig. S15). In Gram-negative bacteria, including most rhizobia, the TonB system provides energy for the TonB-dependent outer membrane receptors to transport iron-porphyrins [55] and plays an important role in the transportation of heme from the external environment [56, 57]. These results suggest that heme or heme-containing proteins act as antidotes of the PBDM interaction between *Streptomyces* sp. FXJ1.4098 and *Mesorhizobium* sp. BAC0120.

To determine whether heme serves as an iron source for *Mesorhizobium* sp. BAC0120, we functionally characterized the role of its heme uptake system in the PBDM interaction. Disruption mutants of the TBDR gene, *hmuU*, and *hmuV* were constructed and subjected to interaction experiments. All mutants failed to grow when co-cultured closely with *Streptomyces* sp. FXJ1.4098, which suggests that the absorption of iron-porphyrin produced by *Streptomyces* sp. FXJ1.4098 is vital for the restoration of *Mesorhizobium* sp. BAC0120 growth (4A). Because all efforts to construct iron-porphyrin-deficient mutants of *Streptomyces* sp. FXJ1.4098 have failed, it seems that the lack of iron-porphyrin biosynthesis is lethal to the strain. Alternatively, hemin, Fe (III)-CP III chloride (coproheme), and hemoglobin were used in combination with DFO to test their growth recovery capacity for *Mesorhizobium* sp. BAC0120 under PBDM-mimic conditions. As expected, the combination of DFO and iron-containing porphyrins or complex successfully induced a PBDM-like phenomenon, confirming iron-porphyrins as the iron source for *Mesorhizobium* sp. BAC0120 in the co-culture (4B). Moreover, the hemin concentration (*c*_hemin_) was found to be positively correlated with the radius of hemin recovery zone (rh), which could be fit by an exponential function (*c*_hemin_ = 0.004374e^0.3521rh^, *R*^2^ = .99; Fig. S16).

**Figure 4:**
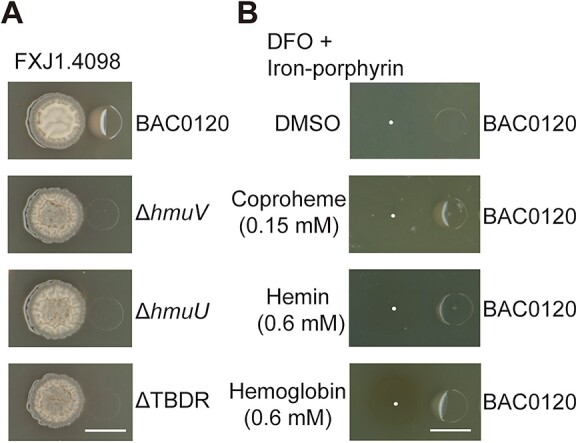
Iron-porphyrin complexes serve as antidotes in PBDM interactions; (A) pairwise interactions between *Streptomyces* sp. FXJ1.4098 and *Mesorhizobium* sp. BAC0120 derivatives (*n* = 3). Δ*hmuV*, Δ*hmuU*, and ΔTBDR represent disruption mutants of *hmuV*, *hmuU*, and TBDR gene in the heme uptake system of *Mesorhizobium* sp. BAC0120, respectively, constructed using the pJQ200SK plasmid; (B) simulation of PBDM phenomenon using mixtures of DFO and iron-porphyrin complexes instead of *Streptomyces* sp. FXJ1.4098 (*n* = 3); first, 20 μl of DFO (20 mM) was mixed with 20 μl of DMSO (solvent control) or iron-porphyrin complex solved in DMSO (0.15 mM for coproheme or 0.6 mM for hemin and hemoglobin); then, the mixture was spotted onto the plate 4 h before the inoculation of *Mesorhizobium* sp. BAC0120. The plate was cultured at 28°C for 4 days; scale, 10 mm.

### PBDM strategy facilitates coexistence of PGPR in the presence of co-isolated bacteria or plant pathogens

To evaluate the ecological significance of the PBDM interaction in the soil, we introduced co-isolated microorganisms with the same sample origin as *Streptomyces* sp. FXJ1.4098 and *Mesorhizobium* sp. BAC0120 for ternary interaction experiments. Eleven co-isolated bacteria belonging to seven genera were tested. In pairwise interactions, eight of the co-isolated bacteria inhibited *Mesorhizobium* sp. BAC0120, and the other three of them coexisted with *Mesorhizobium* sp. BAC0120 (5A and Fig. S17A). Four co-isolated bacteria were inhibited by *Streptomyces* sp. FXJ1.4098, but not FXJ1.4098Δ*desD*, and seven of them coexisted with *Streptomyces* sp. FXJ1.4098 (5B and Fig. S17B). In ternary interactions, *Mesorhizobium* sp. BAC0120 in the vicinity of *Streptomyces* sp. FXJ1.4098 was partially restored even though it was inhibited by co-isolated bacteria in pairwise interactions (5C and Fig. S17C). These results suggest that the PBDM strategy protects *Mesorhizobium* sp. BAC0120 from other antagonistic co-isolated microorganisms.

**Figure 5:**
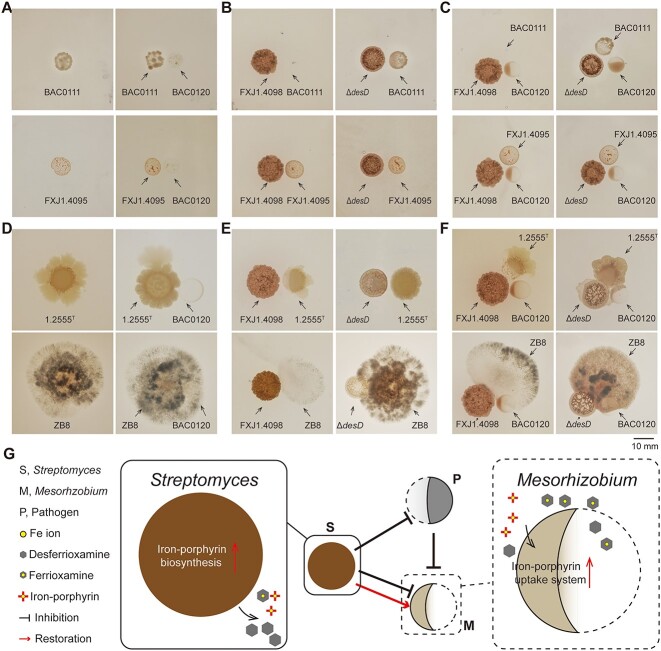
PBDM strategy protects *Mesorhizobium* sp. BAC0120 from antagonistic co-isolated bacteria (A–C) or from plant pathogens (D–F) in tripartite interactions; (A) colonies of representative co-isolated bacteria (left panel) and their pairwise interactions with *Mesorhizobium* sp. BAC0120 (right panel) (*n* = 3); the growth of *Mesorhizobium* sp. BAC0120 was inhibited by the co-isolated bacteria; (B) pairwise interactions between representative co-isolated bacteria and *Streptomyces* sp. FXJ1.4098 or FXJ1.4098Δ*desD* (*n* = 3); the growth of co-isolated bacteria was either inhibited by *Streptomyces* sp. FXJ1.4098 due to its production of DFO (upper) or not affected by *Streptomyces* sp. FXJ1.4098 (lower)*;* (C) tripartite interactions among representative co-isolated bacteria, *Mesorhizobium* sp. BAC0120, and *Streptomyces* sp. FXJ1.4098 or FXJ1.4098Δ*desD* (*n* = 3); the growth of *Mesorhizobium* sp. BAC0120 inhibited by the co-isolated bacteria was partially restored by *Streptomyces* sp. FXJ1.4098; (D) colonies of representative plant pathogens (left panel) and their pairwise interactions with *Mesorhizobium* sp. BAC0120 (right panel) (*n* = 3); the growth of *Mesorhizobium* sp. BAC0120 was inhibited by the plant pathogens; (E) pairwise interactions between representative plant pathogens and *Streptomyces* sp. FXJ1.4098 or FXJ1.4098Δ*desD* (*n* = 3); the growth of the plant pathogens was inhibited by *Streptomyces* sp. FXJ1.4098 partially attributed to its production of DFO; (F) tripartite interactions among representative plant pathogens, *Mesorhizobium* sp. BAC0120, and *Streptomyces* sp. FXJ1.4098 or FXJ1.4098Δ*desD* (*n* = 3); the growth of *Mesorhizobium* sp. BAC0120 inhibited by the plant pathogens was partially restored by *Streptomyces* sp. FXJ1.4098; (G) schematic diagram of PBDM-mediated protection on *Mesorhizobium* sp. BAC0120 by *Streptomyces* sp. FXJ1.4098 in a tripartite co-culture with pathogens; Δ*desD*, DFOE-deficient mutant of *Streptomyces* sp. FXJ1.4098; BAC0111, *Bacillus* sp. BAC0111; FXJ1.4095, *Micromonospora* sp*.* FXJ1.4095; ZB8, *Bipolaris sorokiniana* ZB8; 1.2555^T^, *Agrobacterium rubi* CGMCC 1.2555^T^; scale, 10 mm; for more pictures of tripartite interactions, please refer to Fig. S17.

Since *Streptomyces* and *Mesorhizobium* are known PGPR, we speculated that the protection of *Mesorhizobium* sp. BAC0120 by *Streptomyces* sp. FXJ1.4098 may also function when they encounter plant pathogens. To verify this, we introduced three common plant pathogens: *A. rubi* CGMCC 1.2555^T^, *Bipolaris sorokiniana* ZB8, and *Fusarium oxysporum* f. sp. *cucumerinum* CGMCC 3.2830 into interaction experiments. In the pairwise experiments, the growth of *Mesorhizobium* sp. BAC0120 was inhibited by all the three pathogens (5D and Fig. S17D), whereas *Streptomyces* sp. FXJ1.4098 showed strong inhibitory effects against these pathogens (5E and Fig. S17E). Compared to the wild type, the inhibitory effects of FXJ1.4098Δ*desD* against the above pathogens were remarkably attenuated, indicating the indispensable role of DFOE in the antagonistic bioactivity of *Streptomyces* sp. FXJ1.4098 (5E and Fig. S17E). Similar to the ternary interactions containing co-isolated bacteria, *Mesorhizobium* sp. BAC0120 inhibition by plant pathogens in pairwise interactions was also partially restored in tripartite interactions, suggesting that PBDM-mediated protection on *Mesorhizobium* sp. BAC0120 by *Streptomyces* sp. FXJ1.4098 could function under various conditions (5F and Fig. S17F). These data highlight the potential importance of iron competition and sharing in the maintenance of pathogen-suppressive and plant growth-facilitating microorganisms in the polymicrobial communities (5G).

### PBDM strategy is common between mesorhizobia and red-soil-derived streptomycetes

To explore whether the PBDM interaction occurs between other streptomycetes and rhizobia, we further conducted pairwise interactions between 11 *Streptomyces* strains and seven rhizobial strains (six *Mesorhizobium* strains and *S. meliloti* 2011) (Table S1). Among them, five *Streptomyces* strains isolated from red soil (FXJ1.4033, FXJ1.4034, FXJ1.4038, FXJ1.4098, and FXJ1.172, termed as PBDM-*Streptomyces*, PBDM-S) and all six *Mesorhizobium* strains (BAC0120, BAC0074, CGMCC 1.2541^T^, CGMCC 1.2543^T^, CGMCC 1.11022^T^, and CGMCC 1.12097^T^, termed as PBDM-*Mesorhizobium*, PBDM-M) exhibited the PBDM phenomenon, whereas the other six *Streptomyces* strains displayed either inhibitory or no effects on the growth of rhizobia (6A and Fig. S18). This result suggested that the PBDM strategy is common between *Mesorhizobium* and red-soil-derived *Streptomyces*. Meanwhile, streptomycetes of the same species (*Streptomyces albidoflavus*) displayed similar interaction patterns, although no clear correlation between phylogeny and PBDM could be drawn from the current data (6A). Genome scanning revealed a heme uptake system in all rhizobia used, siderophore biosynthetic gene clusters only in *Mesorhizobium muleiense* CGMCC 1.11022^T^ and *S. meliloti* 2011, and a DFO receptor exclusively in *S. meliloti* 2011. The five PBDM-M strains lacking siderophore biosynthesis and DFO identification capabilities all displayed PBDM interaction with each of the PBDM-S strains, while *M. muleiense* CGMCC 1.11022^T^ that can produce siderophore showed PBDM interaction with only two of the PBDM-S strains (6A). Moreover, *S. meliloti* 2011, with siderophore biosynthesis and DFO utilization capabilities, presented neutral coexistence with the streptomycetes tested (6A). The genomic differences in these rhizobia indicate the importance of siderophore biosynthesis, heme uptake, and DFO utilization capabilities in the PBDM interaction.

**Figure 6:**
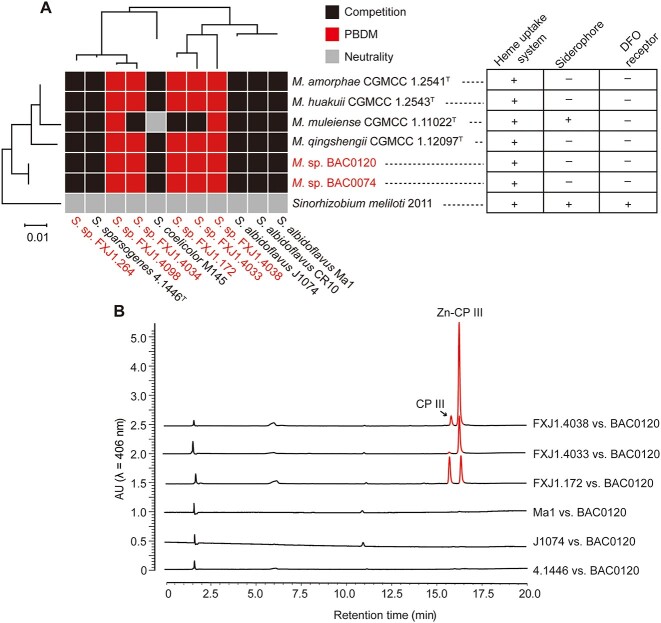
PBDM interactions are common between *Streptomyces* and *Mesorhizobium*; (A) hierarchical clustering heat map of interactions between 11 *Streptomyces* strains and seven rhizobia (*n* = 3); based on the growth of the co-cultured *Streptomyces* and rhizobial colonies, the interactions were determined to be competition, PBDM, and neutrality (see Materials and Methods); representative pictures of the above three interaction types as well as quantitative and statistical analyses of growth inhibition and recovery are given in Fig. S18; the strains isolated from red soil are in red font; maximum-likelihood phylogenetic trees were constructed based on the 16S rRNA gene sequences; the distributions of heme uptake gene clusters, DFOE receptor, and siderophore biosynthetic gene clusters in the seven rhizobial strains are marked on the right; +, homologs detected in the genome; −, no homologs detected; (B) a representative HPLC profile showing detection of CPs in the co-culture plates; CP III and Zn-CP III were detected in the extracts from area b of PBDM co-cultures (FXJ1.4038/FXJ1.4033/FXJ1.172 vs. BAC0120), but not in those of competition co-cultures (J1074/CGMCC 4.1446^T^/Ma1 vs. BAC0120).

To test whether siderophore production is universally involved in interactions between *Streptomyces* and *Mesorhizobium*, a CAS assay was performed to explore the distribution of siderophores in the pairwise interaction plates of *Streptomyces* and *Mesorhizobium* sp. BAC0120. Similar to the plate of FXJ1.4098 versus BAC0120, siderophores were detected in the **a**–**c** areas of all the pairwise plates (Fig. S19). This result suggests that growth inhibition of *Mesorhizobium* by *Streptomyces* is mainly caused by siderophore-mediated iron limitation.

To confirm whether the extracellular porphyrin compounds are related to the observed PBDM interactions, HPLC analyses of extracts from area **b** in the co-culture plates of the streptomycetes versus BAC0120 pairs were conducted. Similar to FXJ1.4098 versus BAC0120, CP III and Zn-CP III were detected in the extracts of the co-cultures with PBDM phenomenon (such as FXJ1.4038 vs. BAC0120, FXJ1.4033 vs. BAC0120, and FXJ1.172 vs. BAC0120), whereas almost no porphyrins were detected in the co-cultures without PBDM phenomenon (such as Ma1 vs. BAC0120, J1074 vs. BAC0120, and CGMCC 4.1446^T^ vs. BAC0120) (6B). This result suggests that the production of extracellular porphyrins may be a common feature of PBDM-S.

## Discussion

We describe an unprecedented PBDM interaction between red-soil-derived *Streptomyces* and *Mesorhizobium*, in which *Streptomyces* protect the nearby *Mesorhizobium* from microbial inhibition. Mutually beneficial coexistence can be widely observed among species with positive interactions, whereas competitive coexistence among species is dependent on their dynamic metabolic adaptation and niche differentiation [58, 59]. PBDM is a complex phenomenon, not limited to classical facilitation (positive) or inhibition (competitive), indicating that microbial interactions are far more complex than previously expected. Phenotypes of *Mesorhizobium* sp. BAC0120 colonies in the PBDM interaction varied according to their distance to *Streptomyces* sp. FXJ1.4098, resulting in spatial structure-dependent coexistence, which is in line with that in the original soil habitat. Red soil has a high content of iron, but most of which is in biologically unavailable iron oxides form. Therefore, microorganisms in the red soil compete with each other intensively for the biologically limited iron resource [19]. All PBDM-S strains were isolated from the red soil [19, 37], whereas streptomycetes from other habitats failed to trigger this interaction. This finding implies that habitat may play an indispensable role in the evolution of inter-phylum interactions. However, a systematic study involving more strains from diverse habitats is needed to determine whether the PBDM phenomenon is widespread between rhizobia and *Streptomyces* derived from other habitats.

We found that PBDM-S and PBDM-M strains displayed complementary metabolic characteristics absent in non-PBDM bacteria. PBDM-S strains are capable of synthesizing and secreting siderophores and iron-porphyrin complexes (heme) into the extracellular environment, whereas PBDM-M strains maintain an iron-porphyrin uptake system instead of siderophore production. DFOs, and perhaps some other siderophores, can scavenge ferric ions from some iron-containing proteins [60], but not from heme or heme-containing proteins [61]. Given that *Streptomyces* produce a wide variety of siderophores [62], other highly diffusible siderophores besides DFOs may also serve as inhibitors in the PBDM interaction. Moreover, we examined 323 soil-derived pre-assembled metagenomes retrieved from Integrated Microbial Genomes and Microbiomes (https://img.jgi.doe.gov) and found that all of them contain heme biosynthesis and heme uptake genes from bacteria (unpublished data). These suggest that the siderophore- and iron-porphyrin-mediated PBDM identified in our study may be an unneglectable component in soil microbial interactions.

Unlike the other five PBDM-M strains, *M*. *muleiense* CGMCC 1.11022^T^ exhibited diverse interaction types with *Streptomyces* (6A), which may be partially attributable to its dual capability of siderophore biosynthesis and iron-porphyrin uptake. Another possible explanation may be that the inducing effects of *M. muleiense* CGMCC 1.11022^T^ on *Streptomyces* iron-porphyrin secretion vary among different *Streptomyces* strains. *Sinorhizobium meliloti* 2011 has outer membrane receptors to recognize and pirate DFOs; hence, it can coexist with DFO-producing *Streptomyces* (6A). Our findings reveal the diverse strategies adopted by co-existing microorganisms to overcome the iron competition in the soil.

Despite attempts for several porphyrin extraction methods [53, 63, 64], we did not detect iron-porphyrin in the extracts even from the medium containing exogenous iron-porphyrin complexes such as hemin. In a recent study on cryptic metabolite-driven exploratory growth of *Streptomyces venezuelae*, the authors also detected the secretion of unmetallated and zinc-bound CPs, but not iron-bound CP or other iron-porphyrins [65]. Therefore, it is likely that iron-porphyrin secreted by *Streptomyces* sp. FXJ1.4098 forms insoluble complexes with the components of the medium, making direct extraction and detection impossible. This may explain why the extracts from area **b** cannot restore the growth of *Mesorhizobium* under iron-limited conditions. Nevertheless, the experiments of heme uptake gene disruption and compound-simulated PBDM provided circumstantial evidence for the release of iron-porphyrin complexes as antidotes from *Streptomyces*.

Few extracellular secretion systems of iron-porphyrin are known in microorganisms, except for the HrtAB system. This system is an ABC heme-dedicated efflux pump that plays a critical role in heme detoxification in many Gram-positive pathogenic and commensal bacteria, including *Corynebacterium diphtheriae* [66], *Lactococcus lactis* [67, 68], and *Staphylococcus aureus* [67, 68]. However, the HrtAB system has not been found in *Streptomyces*. Alternatively, *Streptomyces* can secrete extracellular vesicles that carry many molecules, including bacterioferritin, which stores iron and iron-porphyrins [69, 70]. Also, Wang *et al*. discovered that a *Dietzia* strain achieves cross-species iron source delivery via vesicles [71]. Therefore, we speculate that the delivery of iron-porphyrins during PBDM may be mediated by vesicles. However, isolation of vesicles from co-culture plates remains challenging. We thus isolated and purified the vesicles of *Streptomyces* sp. FXJ1.4098 cultured in liquid medium, but these vesicles failed to restore the growth of *Mesorhizobium* sp. BAC0120 under iron-limited conditions (data now shown). Furthermore, to confirm whether bacterioferritin is involved in PBDM, we constructed a bacterioferritin deletion mutant of *Streptomyces* sp. FXJ1.4098, but the mutant still retained PBDM interaction with *Mesorhizobium* sp. BAC0120 (data not shown). These results suggest that vesicles from *Streptomyces* sp. FXJ1.4098 may not induce PBDM, although the contribution of vesicles to PBDM in the solid plates cannot be ruled out. In contrast, the iron-porphyrin-containing protein bovine hemoglobin relieved the growth inhibition of DFOB on *Mesorhizobium* sp. BAC0120 (Fig. 3B), suggesting that hemoproteins secreted by *Streptomyces* sp. FXJ1.4098 are likely the antidote in PBDM. Hence, future analysis of the extracellular proteome in the co-culture to identify proteins with iron-porphyrin cofactors is warranted. In addition, the construction and screening of random mutation libraries of PBDM-S strains for non-PBDM mutants would also help to elucidate the mechanism of iron-porphyrin delivery among bacteria.

Understanding interspecies interactions in complex polymicrobial coexistence is important for the utilization of PGPR to synergistically facilitate plant growth and inhibit phytopathogens. This is exemplified by the growth-facilitating effect of *M. ciceri* on chickpeas, which is greatly enhanced when co-inoculated with *Streptomyces* [8]. However, the interaction between *Mesorhizobium* and *Streptomyces* and the mechanism underlying their coexistence are not known. Our findings demonstrate that *Mesorhizobium* species can coexist with antimicrobial streptomycetes via the PBDM strategy. Moreover, the PBDM strategy protects nonantagonistic PGPR from antagonistic microorganisms including plant pathogens (5 and Fig. S17), which provides insights into the ecological implications of this unprecedented interaction strategy. That is, by *Streptomyces* conferring benefit upon *Mesorhizobium*, they may synergistically promote the growth of plant that they are living on. The plant may in turn increase the fitness of *Streptomyces* by providing nutrients through root exudates. This may explain how protecting other PGPR from microbial competition via PBDM is beneficial to streptomycetes themselves. These results highlight the potential role of *Streptomyces* in regulating rhizosphere communities, which usually harbor both PGPR and pathogens. Nevertheless, further experiments are required to elucidate the functional significance of the PBDM interaction for host plant growth and health.

## Conclusion

In this study, we demonstrated an unprecedented and widespread PBDM interaction between *Streptomyces* and *Mesorhizobium* via sequestration and release of iron. We found that *Streptomyces* inhibited the growth of distant *Mesorhizobium* by creating an iron-deficient environment via DFO secretion. *Streptomyces* also released iron-porphyrin complexes around its colonies, thereby restoring the growth of nearby *Mesorhizobium*. Ternary interactions showed that *Streptomyces* simultaneously inhibited both *Mesorhizobium* and plant pathogens indiscriminately via iron sequestration by DFO, but spared the nearby *Mesorhizobium* via iron sharing by iron-porphyrin complexes. These findings suggest that the PBDM strategy facilitates the coexistence of diverse pathogen-suppressive and plant growth-facilitating microorganisms in the soil.

## Supplementary Material

Supplements-final_wrad041

## Data Availability

Whole genome sequencing data of *Mesorhizobium* sp. BAC0120 and *Streptomyces* sp. FXJ1.4098 are available in the NCBI BioProject PRJNA1035288 and PRJNA1035290, respectively. The RNA-seq data of *Streptomyces* sp. FXJ1.4098 are available in the NCBI BioProject PRJNA1036711. Other data generated in this study are provided in the text and the Supplementary Material.
