# Supplementary material for: Proximity-based defensive mutualism between Streptomyces and Mesorhizobium by sharing and sequestering iron
Source: ISME J. 2024 Jan 10;18(1):wrad041. doi: 10.1093/ismejo/wrad041 (PMC10881299; doi:10.1093/ismejo/wrad041)
Supplement: Supplements-final_wrad041 [file supplements-final_wrad041.pdf]

Supplementary Material

Proximity-based defensive mutualism between *Streptomyces* and *Mesorhizobium* by sharing and sequestering iron

Running head: Proximity-based defensive mutualism

Xueyuan Du[1,2,3], Ning Liu[1], Bingfa Yan[1,2], Yisong Li[4], Minghao Liu[1*] and Ying Huang[1,2*]

[1]*State Key Laboratory of Microbial Resources, Institute of Microbiology, Chinese Academy of Sciences, Beijing 100101, P. R. China.*

[2]*College of Life Sciences, University of Chinese Academy of Sciences, Beijing 101408, P. R. China.*

[3]*National Engineering Laboratory for Site Remediation Technologies, BCEG Environmental Remediation Co., Ltd., Beijing 100015, P. R. China.*

[4]*School of Public Health, Qingdao University, Qingdao 266071, P. R. China.*

[*]**Correspondence:**

Minghao Liu, *State Key Laboratory of Microbial Resources, Institute of Microbiology, Chinese Academy of Sciences, NO.1 Beichen West Road, Chaoyang District, Beijing 100101, P. R. China; Email: lysf1987313@163.com.*

Ying Huang, *State Key Laboratory of Microbial Resources, Institute of Microbiology, Chinese Academy of Sciences, NO.1 Beichen West Road, Chaoyang District, Beijing 100101, P. R. China; Email: huangy@im.ac.cn.*

Supplementary Figures

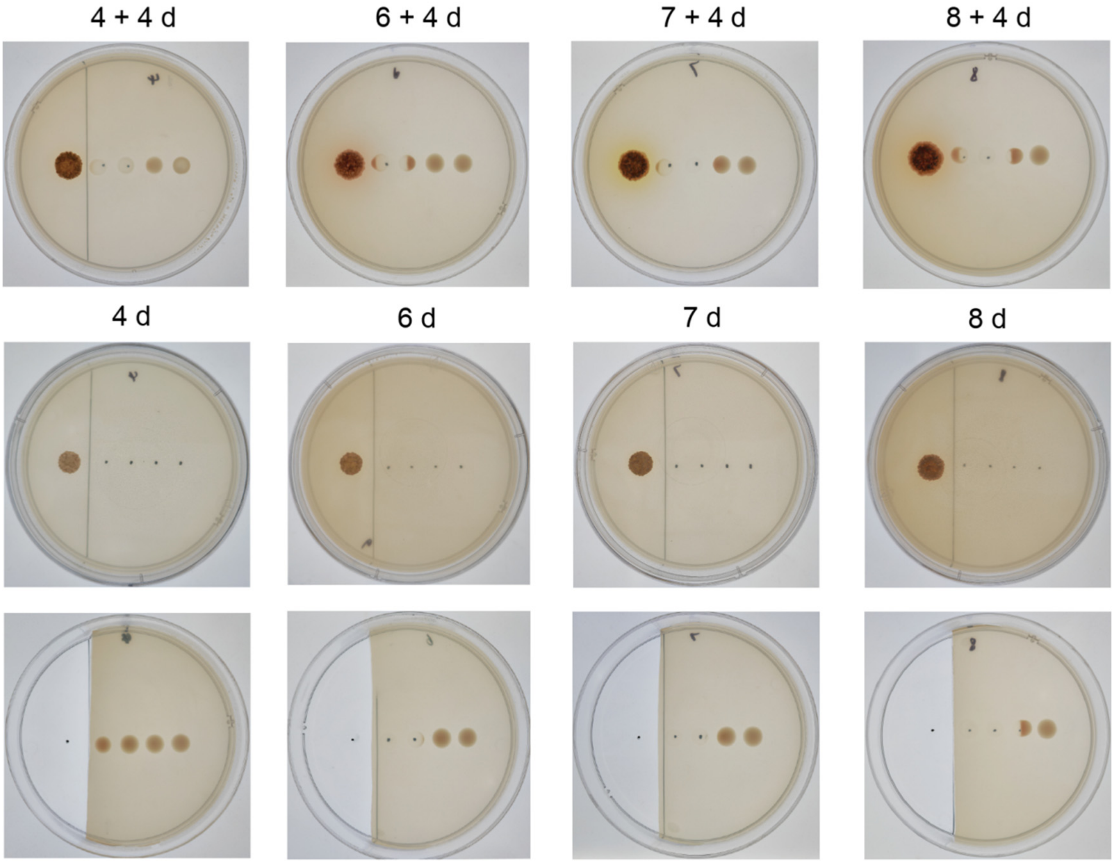

Fig. S1. Representative images showing interaction between *Streptomyces* sp. FXJ1.4098 and *Mesorhizobium* sp. BAC0120 after removal of pre-monocultured *Streptomyces* sp. FXJ1.4098.

No **p**roximity-**b**ased **d**efensive **m**utualism (PBDM)-like phenomenon could be observed when *M.* sp. BAC0120 was cultured for four days on a plate after removal of *Streptomyces* pre-cultured for four to eight days (n = 3 biologically independent experiments).

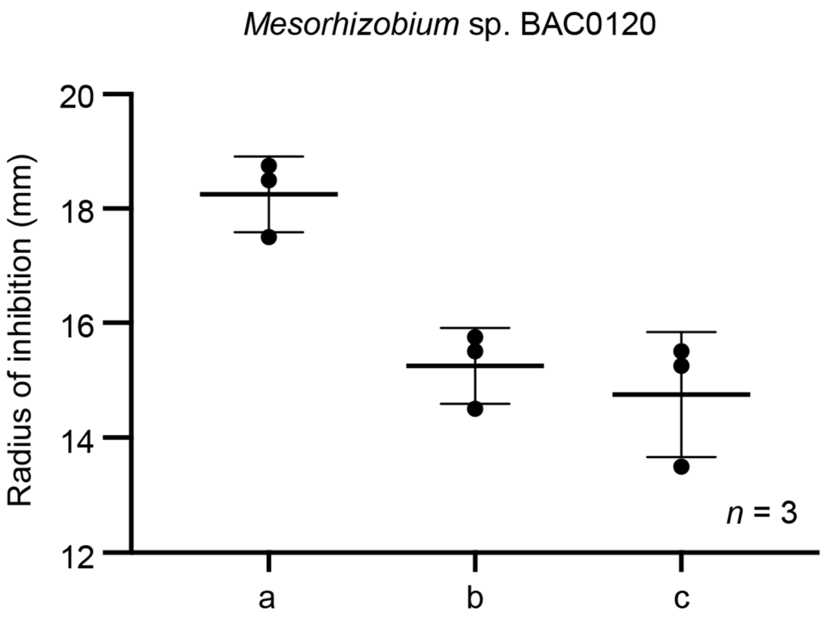

Fig. S2. Quantification of inhibitory activity on *M.* sp. BAC0120 of the extracts from interaction plate areas a, b, and c shown in Fig. 2B.

The inhibitory activity was quantified by measuring the radius of the inhibition zone. Results are shown as dot plots and depicted with mean ± standard deviation.

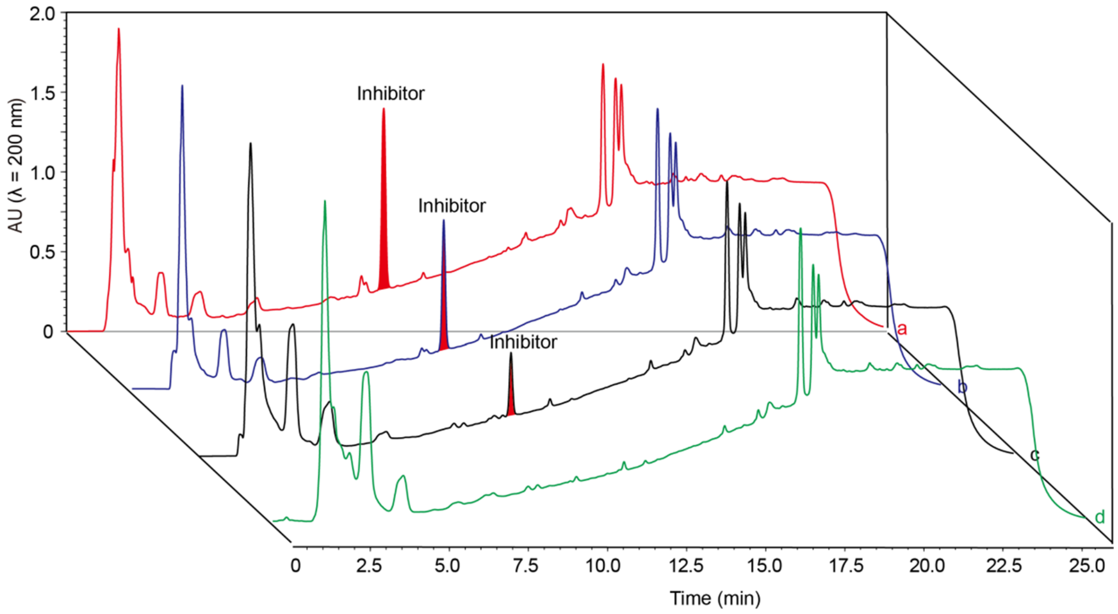

Fig. S3. A representative HPLC profile showing a peak of inhibitor as the only remarkably differential metabolite distinguishing extracts from areas a−d in the co-culture plate of *S*. sp. FXJ1.4098 vs. *M*. sp. BAC0120 (*n* = 3).

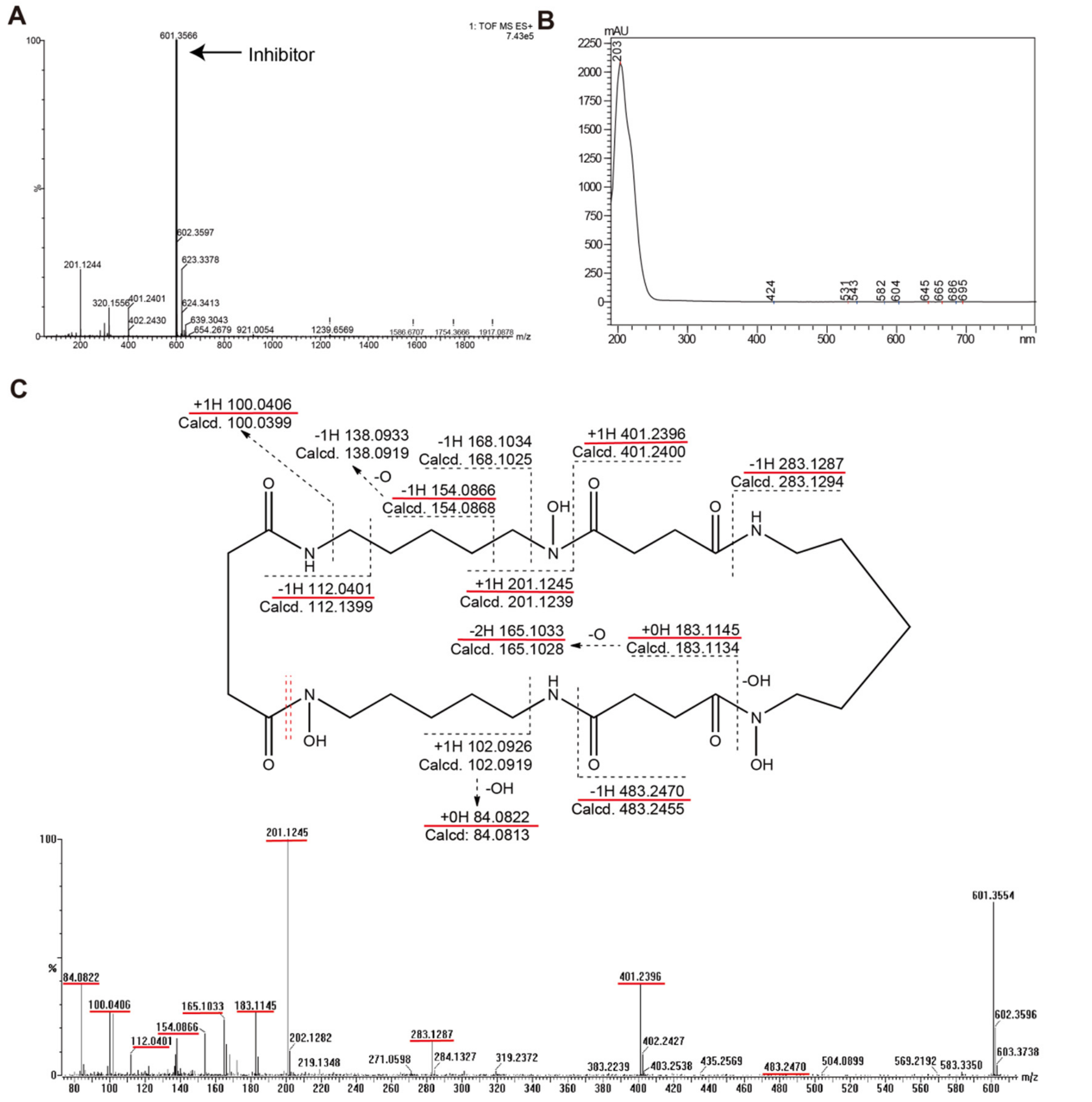

Fig. S4. Chemical elucidation and identification of the inhibitor.

A. Mass spectrum of the inhibitor. **B.** Ultraviolet (UV) spectrum of the inhibitor. The mass signal of the inhibitor was at *m/z* 601.3566 $[M + H]^+$ and it only displayed an end UV absorbance. **C.** Annotation of the CID MS/MS spectrum for the $[M + H]^+$ ion of the inhibitor. Peaks underlined in red are consistent with the fragmentation pattern proposed for desferrioxamine E (DFOE) in the top panel, which assumes a macrocycle opening at the position of the red double dotted line.

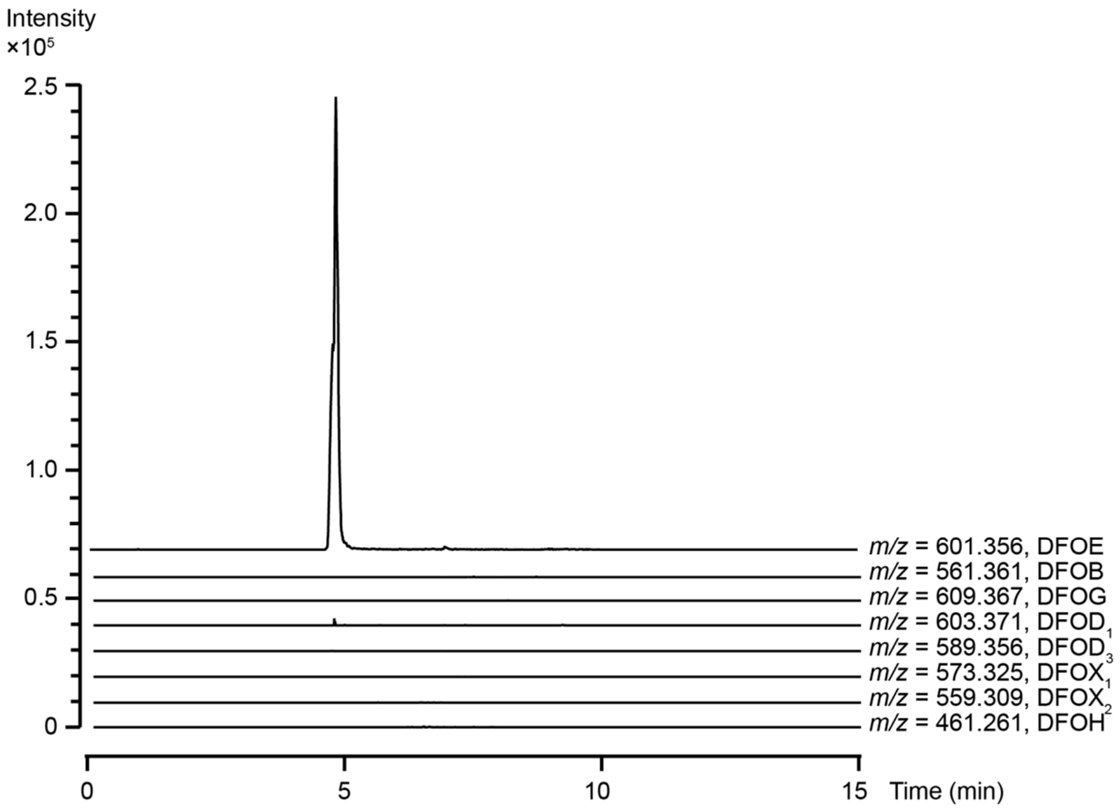

Fig. S5. Extracted ion chromatograms from UHPLC-HRMS analysis of culture extracts from *S*. sp. FXJ1.4098.

The results of both the monoculture and coculture were the same. The *m/z* values of desferrioxamines (DFOs) used to generate each chromatogram are listed on the right.

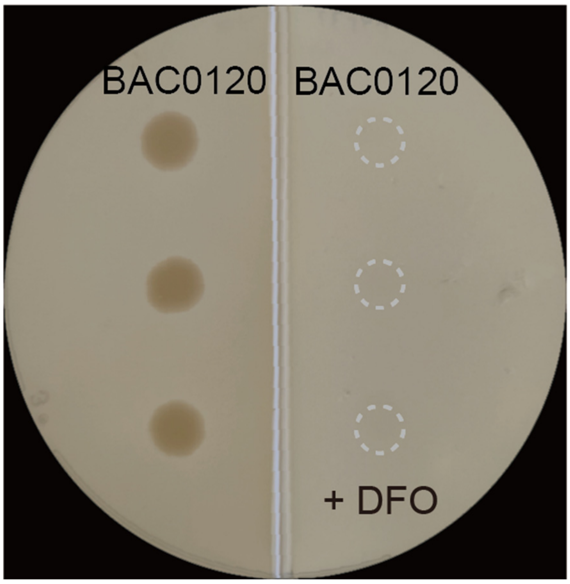

Fig. S6. A representative image showing the growth inhibition effect of desferrioxamine (DFO) on *M.* sp. BAC0120.

Growth comparison of *M.* sp. BAC0120 on medium with or without 200 μM DFOB (*n* = 3). *M.* sp. BAC0120 was spotted onto GYM agar (left) and GYM agar containing 200 μM DFOB (right). The photograph was taken four days after inoculation of *M.* sp. BAC0120. The white dotted circle indicates the original inoculation sites of *M.* sp. BAC0120.

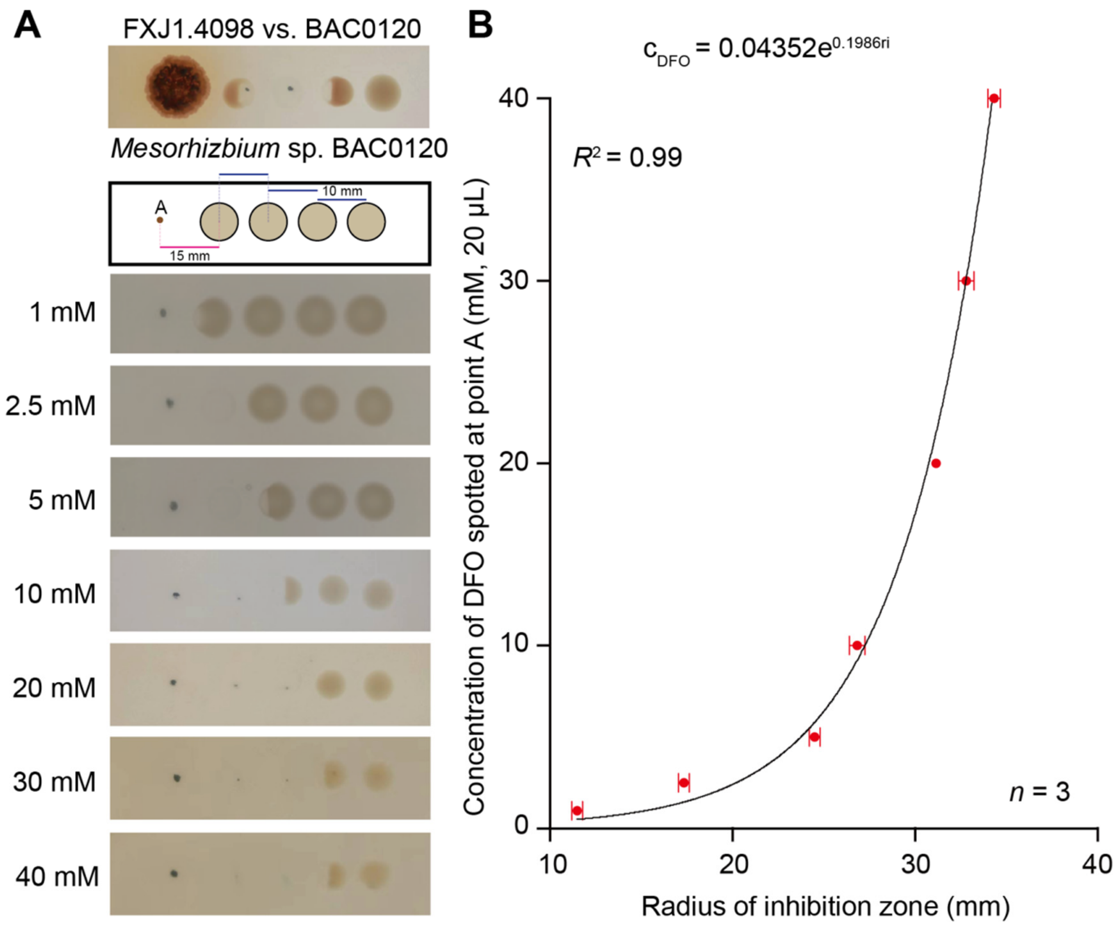

Fig. S7. A dynamic model of DFO diffusivity.

A. Representative images showing the inhibition of 20 μL of DFO with different concentrations against *M.* sp. BAC0120. DFO was spotted at point A. The growth of *M.* sp. BAC0120 was recorded after 4 days of culture. **B.** The radius of the inhibition zone and the concentration of DFO conform to an exponential fitting function: $c_{DFO}=0.04352e^{0.1986ri}$. "$c_{DFO}$" represents the concentration of DFO spotted at point A; "e" indicates Euler number; and "ri" indicates the inhibition zone radius against *M.* sp. BAC0120.

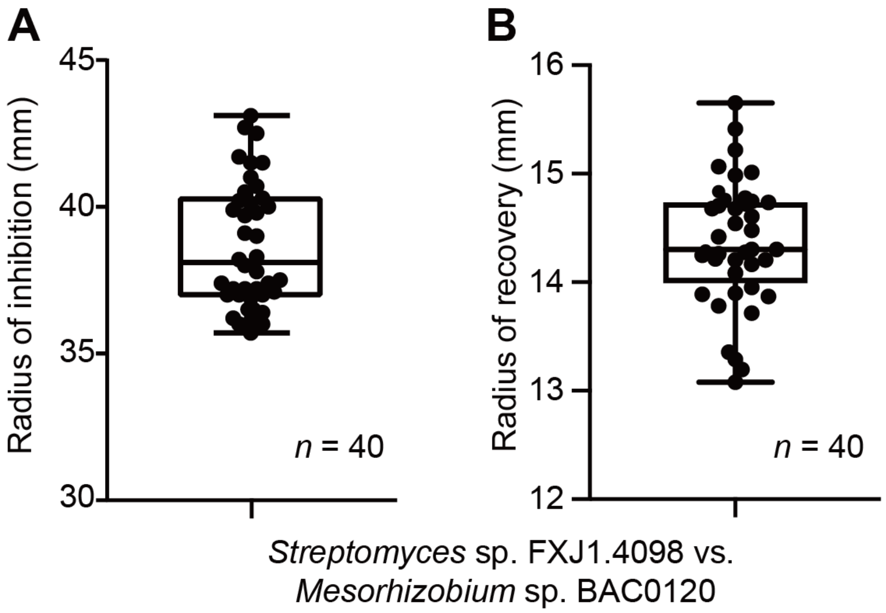

Fig. S8. Quantification of growth inhibition and recovery of *M.* sp. BAC0120 by *S.* sp. FXJ1.4098.

A. The inhibitory activity of *S.* sp. FXJ1.4098 was quantified by measuring the radius of inhibition zone around the colony. **B.** The growth recovery activity of *S.* sp. FXJ1.4098 was quantified by calculating the radius of recovery zone (the zone where *M*. sp. BAC0120 can grow around *S.* sp. FXJ1.4098). Results are shown as box plots based on 40 biologically independent experiments. The center horizontal line denotes the median and boxes extend from the 25th to the 75th percentile of values. Whiskers show the full range of value distribution.

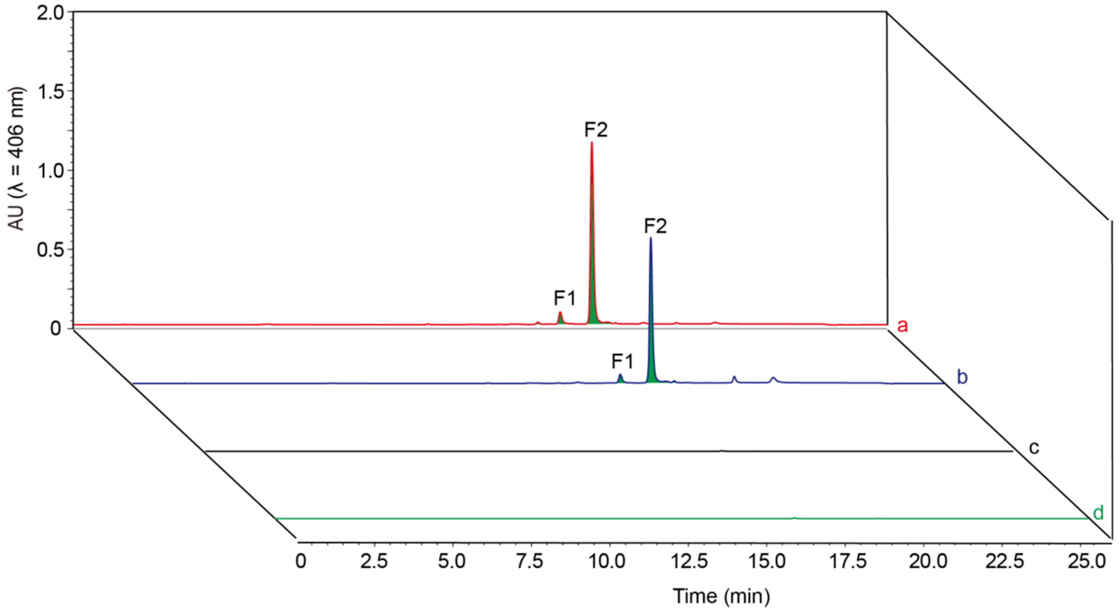

Fig. S9. HPLC detection and identification of differential compounds in the extracts from areas a and b of co-culture plates of *S.* sp. FXJ1.4098 vs. *M.* sp. BAC0120

At the absorption value of 406 nm, F1 and F2 can be obviously detected in the extracts from areas **a** and **b** (Fig. 2A) of the co-culture plate, but not areas **c** and **d** ($n = 3$).

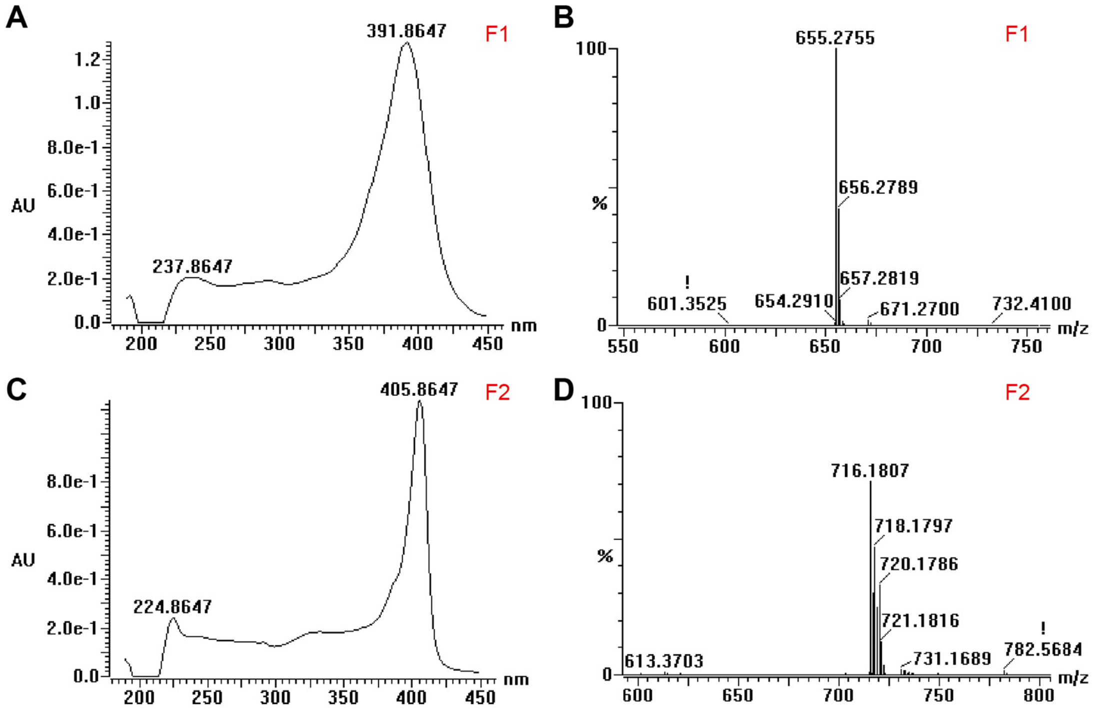

Fig. S10. UV and mass spectra of Product F1 and F2.

A. UV spectrum of product F1. The maximum UV absorbance was at 392 nm. **B.** Mass spectrum of product F1. The mass signal of product F1 was at *m/z* 655.2755 $[M + H]^+$. **C.** UV spectrum of product F2. The maximum UV absorbance was at 406 nm. **D.** Mass spectrum of product F2. The mass signal of product F2 was at *m/z* 716.1807 $[M + H]^+$.

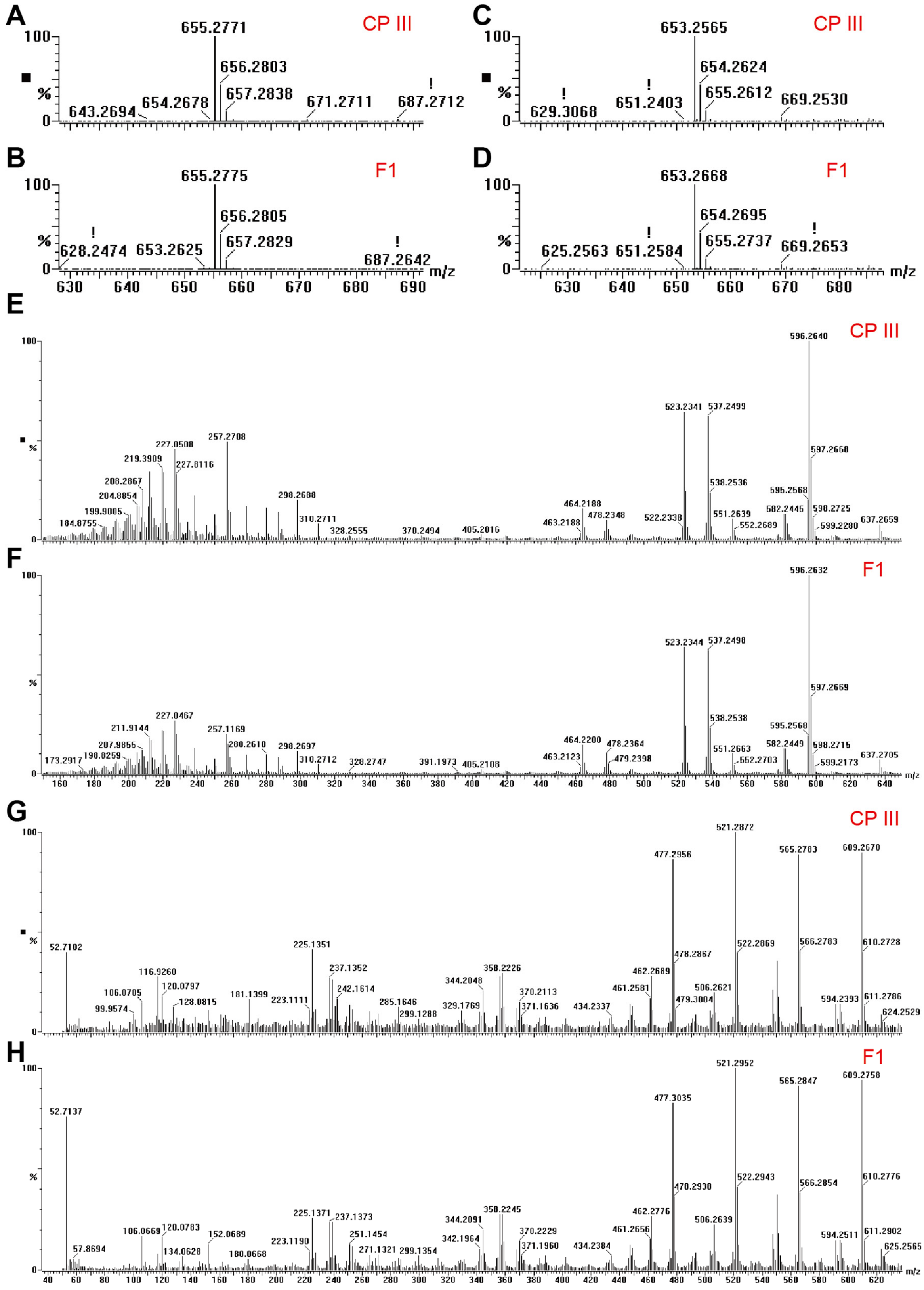
A
CP III
655.2771
656.2803
657.2838
643.2694
654.2678
671.2711
687.2712
B
F1
655.2775
656.2805
657.2829
628.2474
653.2625
687.2642
630 640 650 660 670 680 690
m/z
C
CP III
653.2565
654.2624
655.2612
629.3068
651.2403
669.2530
D
F1
653.2668
654.2695
655.2737
625.2563
651.2584
669.2653
630 640 650 660 670 680
m/z
E
CP III
596.2640
523.2341
537.2499
597.2668
538.2536
595.2568
257.2708
227.0508
219.3909
227.8116
F
F1
596.2632
523.2344
537.2498
597.2669
538.2538
227.0467
211.9144
257.1169
G
CP III
521.2872
477.2956
565.2783
609.2670
522.2869
566.2783
610.2728
225.1351
H
F1
521.2952
565.2847
609.2758
477.3035
52.7137
225.1371
m/z

Fig. S11. UHPLC-HRMS profiling of F1 matches that of coproporphyrin III (CP III).

A and **B.** Positive mode mass spectra of CP III and F1. The mass signals of CP III and F1 were at *m/z* 655.2775 [M + H]$^+$ and *m/z* 655.2771 [M + H]$^+$, respectively. **C** and **D.** Negative mode mass spectra of CP III and F1. The mass signals of CP III and F1 were at *m/z* 653.2565 [M - H]$^-$ and *m/z* 653.2668 [M - H]$^-$, respectively. **E** and **F.** Positive model MS/MS spectra of CP III and F1, respectively. **G** and **H.** Negative model MS/MS spectra of CP III and F1, respectively.

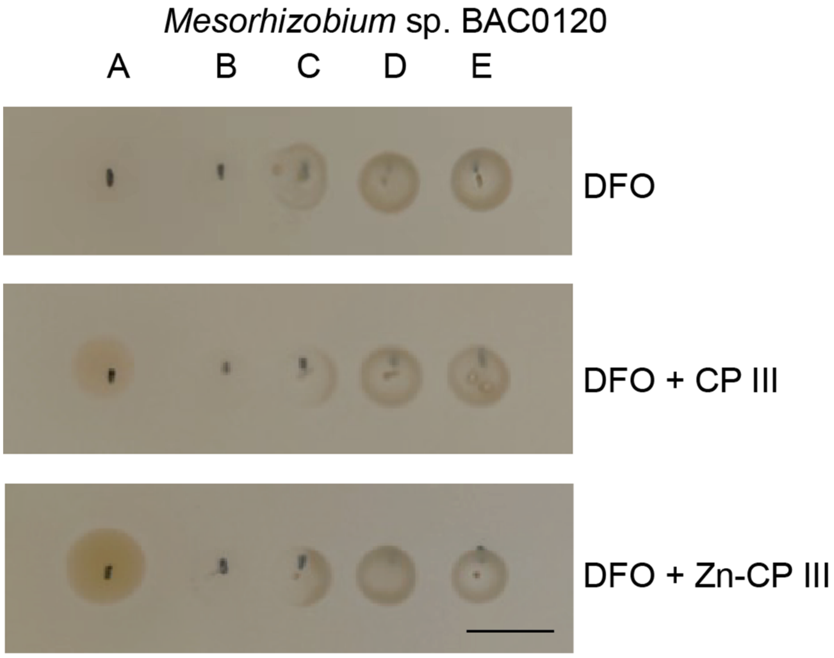

Fig. S12. Detection of the growth restoring effects of products F1 (CP III) and F2 (Zn-CP III) on *M*. sp. BAC0120 (*n* = 3).

A volume of 20 µL of DFOB (20 mM) was mixed with 20 µL of F1 (5 mM) or F2 (5 mM) to form a premix, which was then spotted at point A on the plate. After 4 h, *M*. sp. BAC0120 was drop plated at points B to E and then further cultured at 28℃ for four days. Scale, 10 mm.

115

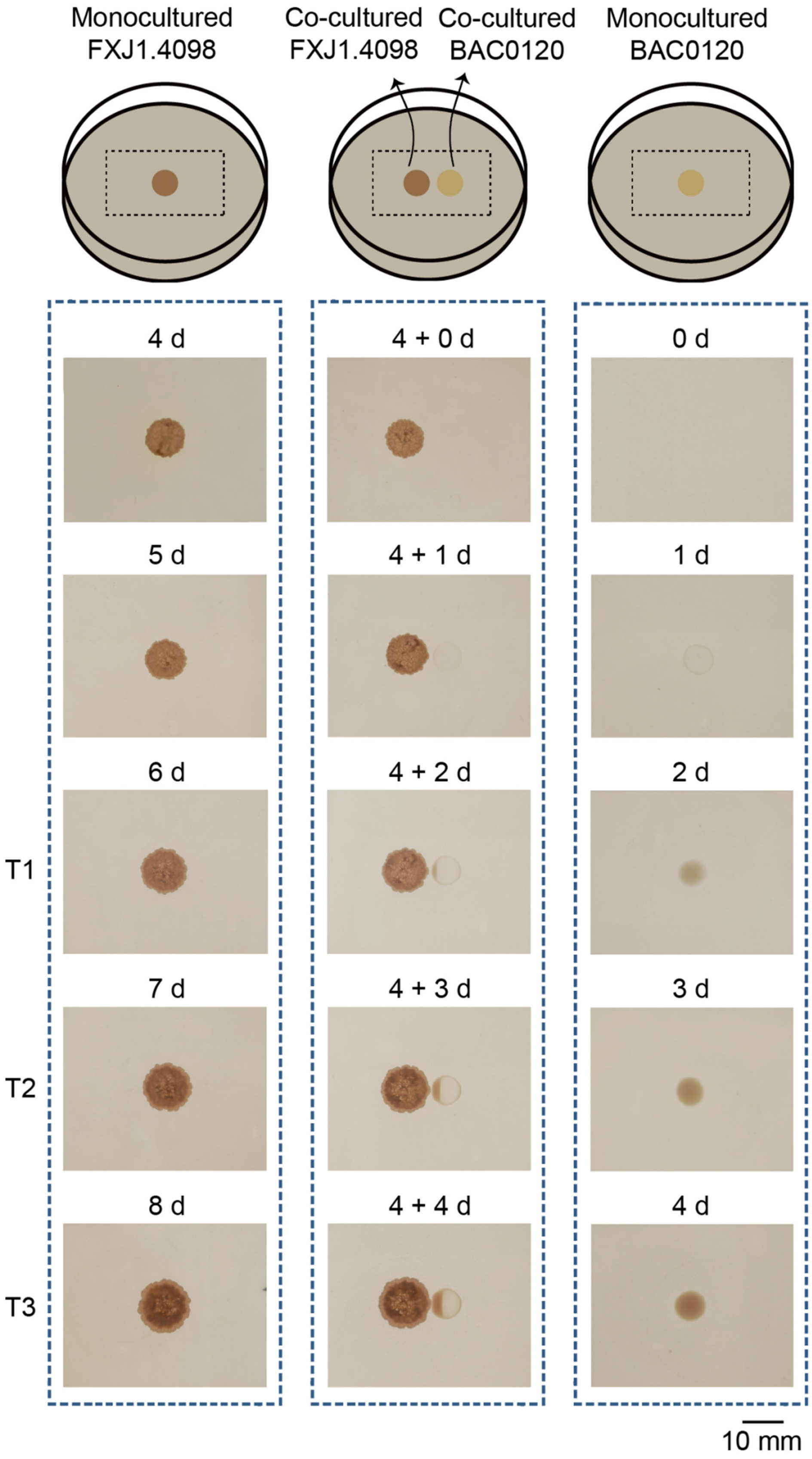

Monocultured FXJ1.4098
Co-cultured FXJ1.4098
Co-cultured BAC0120
Monocultured BAC0120
4 d
5 d
6 d
7 d
8 d
4 + 0 d
4 + 1 d
4 + 2 d
4 + 3 d
4 + 4 d
0 d
1 d
2 d
3 d
4 d
T1
T2
T3
10 mm

Fig. S13. Transcriptome and RT-qPCR sampling of *S*. sp. FXJ1.4098 and *M*. sp. BAC0120 under monoculture and co-culture conditions.

In the co-culture plates, *S*. sp. FXJ1.4098 was inoculated four days ahead of *M*. sp. BAC0120. Sampling of *S*. sp. FXJ1.4098 was performed after its inoculation for six days (T1) and seven days (T2). Sampling of *M*. sp. BAC0120 was performed after its inoculation for three days (T2) and four days (T3).

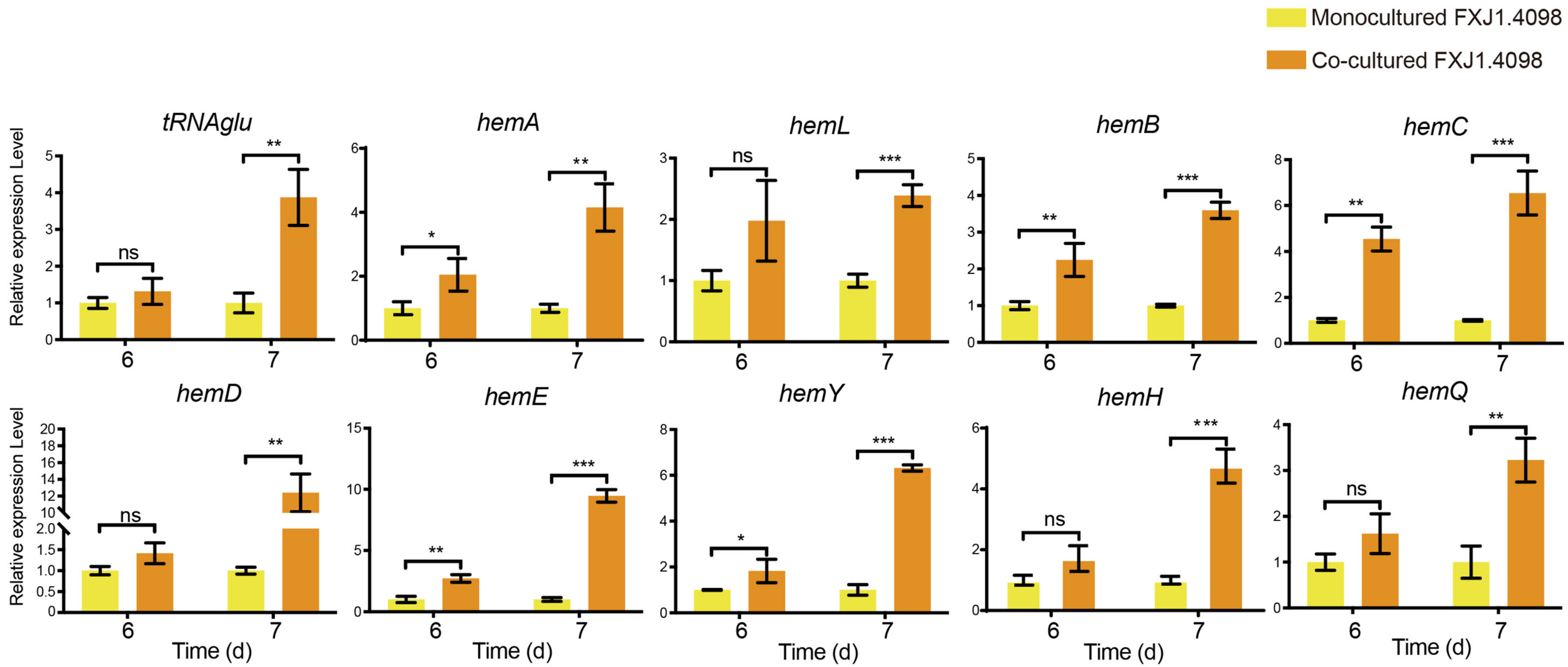

Fig. S14. Transcriptional analyses of heme biosynthetic genes in *S*. sp. FXJ1.4098 by RT-qPCR.

Total RNAs were isolated from *S*. sp. FXJ1.4098 monocultured or co-cultured for six and seven days, and used for synthesizing cDNA. The 16S rRNA gene was used as an internal reference to normalize the RNA concentration. The relative transcription levels of 10 genes (*tRNAglu, hemA, hemL, hemB, hemC, hemD, hemE, hemY, hemH*, and *hemQ*) were obtained after normalization against the internal reference at corresponding time points. Error bars show the standard deviation of three independent experiments. Statistical significance was determined by Student's t-test. *, $p < 0.05$; **, $p < 0.01$; ***, $p < 0.001$; ns, not significant.

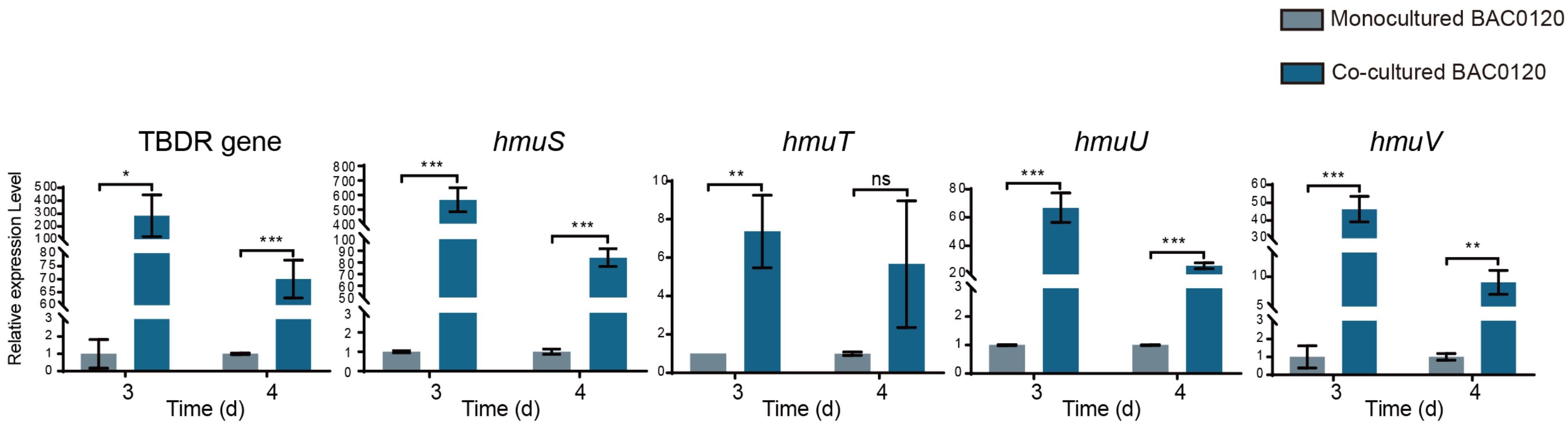

Fig. S15. Transcriptional analyses of heme uptake cluster genes in *M*. sp. BAC0120 by RT-qPCR.

Total RNAs were isolated from *M*. sp. BAC0120 monocultured or co-cultured for three and four days, and used for synthesizing cDNA. The 16S rRNA gene was used as an internal reference to normalize the RNA concentration. The relative transcription levels of 5 genes (TBDR gene, *hmuS, hmuT, hmuU*, and *hmuV*) were obtained after normalization against the internal reference at corresponding time points. Error bars show the standard deviation of three independent experiments. Statistical significance was determined by Student's t-test. *, $p < 0.05$; **, $p < 0.01$; ***, $p < 0.001$; ns, not significant.

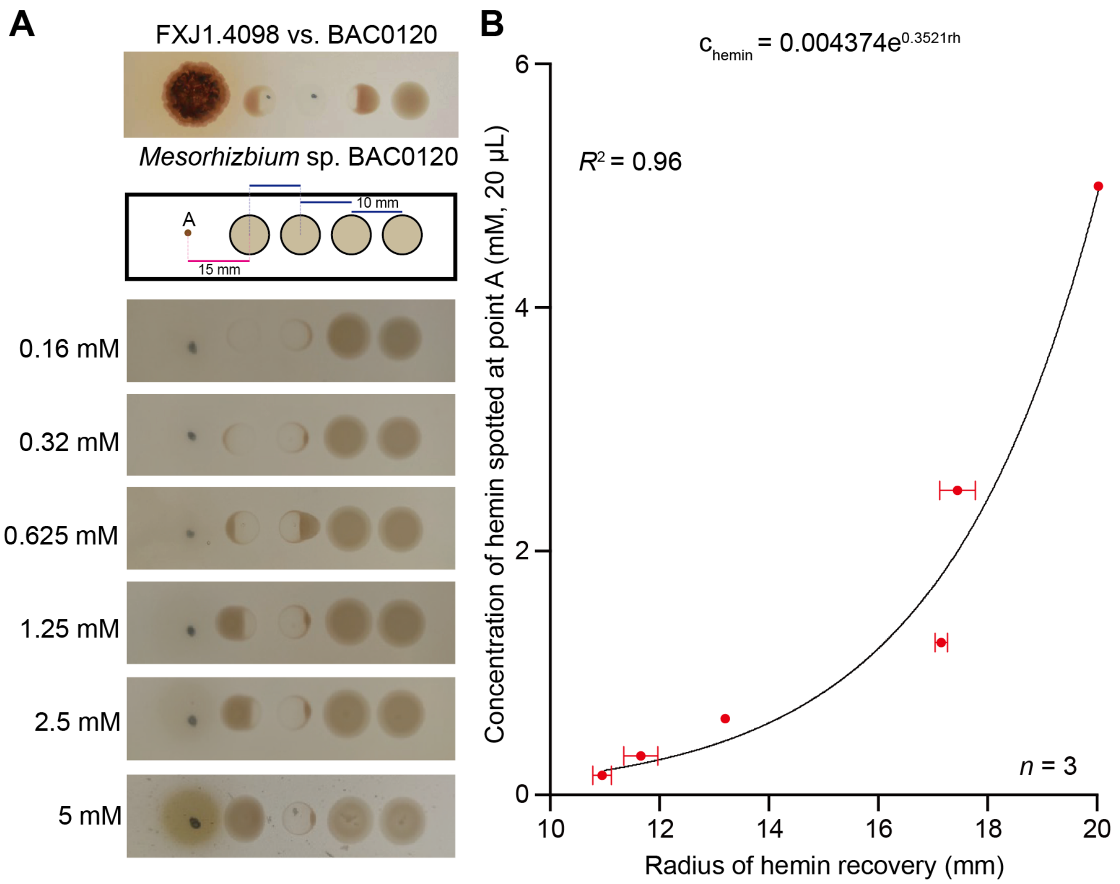

Fig. S16. A dynamic model of hemin diffusivity.

A. Representative images showing growth recovery effect on *M.* sp. BAC0120 by a volume of 20 µL of DFO (20 mM) mixed with 20 µL of hemin in different concentrations. The mixture was spotted at point A. The growth of *M.* sp. BAC0120 was recorded after 4 days of culture. **B.** The radius of hemin recovery (rh, equaled to the radius of recovery zone where *M.* sp. BAC0120 can grow around hemin) and the concentrations of hemin (c_{hemin}) conform to an exponential fitting function: $c_{hemin}=0.004374e^{0.3521rh}$. "$c_{hemin}$" represents the concentration of hemin spotted at point A; "e" indicates Euler number; and "rh" indicates the radius of hemin diffusion.

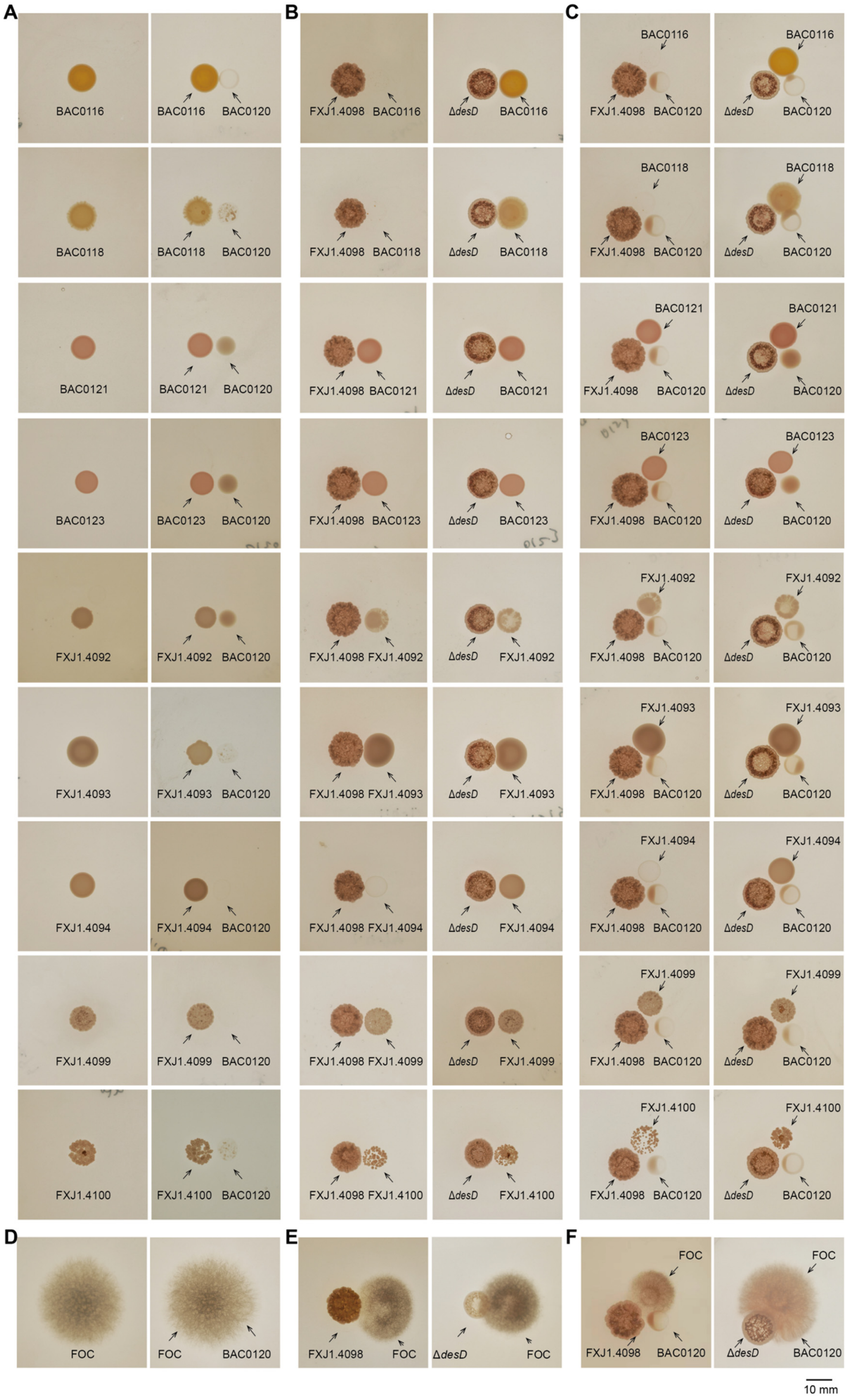
A
BAC0116
BAC0116 BAC0120
BAC0118
BAC0118 BAC0120
BAC0121
BAC0121 BAC0120
BAC0123
BAC0123 BAC0120
FXJ1.4092
FXJ1.4092 BAC0120
FXJ1.4093
FXJ1.4093 BAC0120
FXJ1.4094
FXJ1.4094 BAC0120
FXJ1.4099
FXJ1.4099 BAC0120
FXJ1.4100
FXJ1.4100 BAC0120
B
FXJ1.4098 BAC0116
ΔdesD BAC0116
FXJ1.4098 BAC0118
ΔdesD BAC0118
FXJ1.4098 BAC0121
ΔdesD BAC0121
FXJ1.4098 BAC0123
ΔdesD BAC0123
FXJ1.4098 FXJ1.4092
ΔdesD FXJ1.4092
FXJ1.4098 FXJ1.4093
ΔdesD FXJ1.4093
FXJ1.4098 FXJ1.4094
ΔdesD FXJ1.4094
FXJ1.4098 FXJ1.4099
ΔdesD FXJ1.4099
FXJ1.4098 FXJ1.4100
ΔdesD FXJ1.4100
C
BAC0116 FXJ1.4098 BAC0120
BAC0116 ΔdesD BAC0120
BAC0118 FXJ1.4098 BAC0120
BAC0118 ΔdesD BAC0120
BAC0121 FXJ1.4098 BAC0120
BAC0121 ΔdesD BAC0120
BAC0123 FXJ1.4098 BAC0120
BAC0123 ΔdesD BAC0120
FXJ1.4092 FXJ1.4098 BAC0120
FXJ1.4092 ΔdesD BAC0120
FXJ1.4093 FXJ1.4098 BAC0120
FXJ1.4093 ΔdesD BAC0120
FXJ1.4094 FXJ1.4098 BAC0120
FXJ1.4094 ΔdesD BAC0120
FXJ1.4099 FXJ1.4098 BAC0120
FXJ1.4099 ΔdesD BAC0120
FXJ1.4100 FXJ1.4098 BAC0120
FXJ1.4100 ΔdesD BAC0120
D
FOC
FOC BAC0120
E
FXJ1.4098 FOC
ΔdesD FOC
F
FOC FXJ1.4098 BAC0120
FOC ΔdesD BAC0120
10 mm

Fig. S17. Interactions among *S.* sp. FXJ1.4098, *M.* sp. BAC0120, and co-isolated bacteria, or plant pathogens.

A. Colonies of co-isolated bacteria (left panel) and their pairwise interactions with *M.* sp. BAC0120 (right panel) (*n* = 3). The growth of *M*. sp. BAC0120 was inhibited or not affected by the co-isolated bacteria. **B.** Pairwise interactions between co-isolated bacteria and *S*. sp. FXJ1.4098 or FXJ1.4098Δ*desD* (*n* = 3). The growth of co-isolated bacteria was either inhibited by *S.* sp. FXJ1.4098 due to its production of DFO or not affected by *S.* sp. FXJ1.4098. **C.** Tripartite interactions among *M.* sp. BAC0120, co-isolated bacteria, and *S*. sp. FXJ1.4098 or FXJ1.4098Δ*desD* (*n* = 3). The growth of *M*. sp. BAC0120 inhibited by the co-isolated bacteria was partially restored by *S.* sp. FXJ1.4098, and the growth of *M*. sp. BAC0120 not affected by the co-isolated bacteria was partially inhibited by *S.* sp. FXJ1.4098. **D.** The colony of a plant pathogen (left panel) and its pairwise interaction with *M.* sp. BAC0120 (right panel) (*n* = 3). The growth of *M.* sp. BAC0120 was inhibited by the plant pathogen. **E.** Pairwise interactions between a plant pathogen and *S*. sp. FXJ1.4098 or FXJ1.4098Δ*desD* (*n* = 3). The growth of the plant pathogen was partially inhibited by *S.* sp. FXJ1.4098 attributed to its production of DFO. **F.** Tripartite interactions among a plant pathogen, *M.* sp. BAC0120, and *S*. sp. FXJ1.4098 or FXJ1.4098Δ*desD* (*n* = 3). The growth of *M*. sp. BAC0120 inhibited by the plant pathogen was partially restored by *S*. sp. FXJ1.4098. BAC0116, *Massilia* sp. BAC0116; BAC0118, *Massilia* sp. BAC0118; BAC0121, *Microvirga* sp. BAC0121; BAC0123, *Noviherbaspirillum* sp. BAC0123; FXJ1.4092, *Arthrobacter* sp. FXJ1.4092; FXJ1.4093, *Arthrobacter* sp. FXJ1.4093; FXJ1.4094, *Arthrobacter* sp. FXJ1.4094; FXJ1.4099, *Streptomyces* sp. FXJ1.4099; FXJ1.4100, *Streptomyces* sp. FXJ1.4100; FOC, *Fusarium oxysporum* f. sp. *cucumerinum* CGMCC 3.2830; Δ*desD*, DFOE-deficient mutant of *S.* sp. FXJ1.4098. Scale, 10 mm.

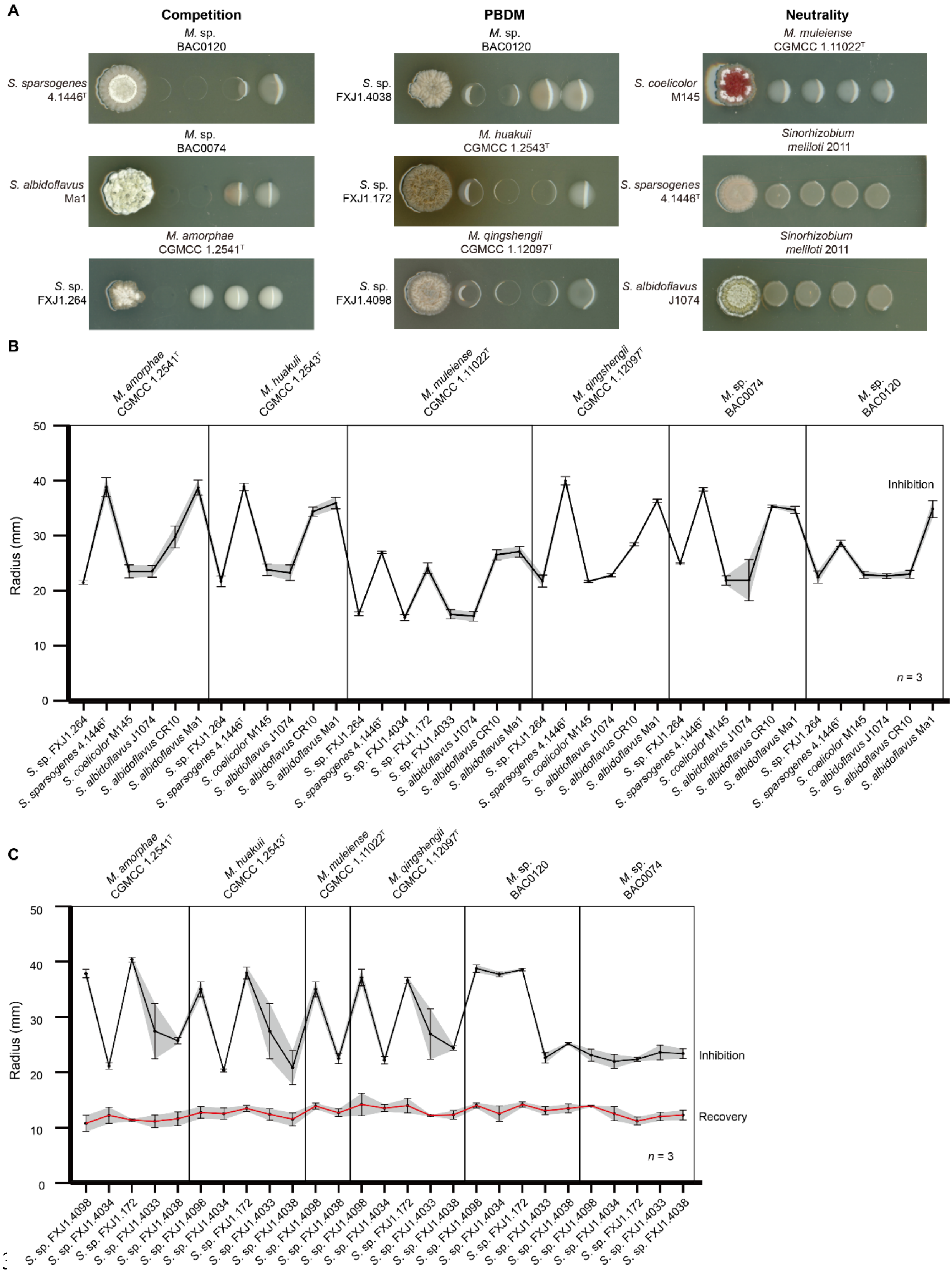
A
Competition
PBDM
Neutrality
M. sp. BAC0120
S. sparsogenes 4.1446T
M. sp. BAC0074
S. albidoflavus Ma1
M. amorphae CGMCC 1.2541T
S. sp. FXJ1.264
M. sp. BAC0120
S. sp. FXJ1.4038
M. huakuii CGMCC 1.2543T
S. sp. FXJ1.172
M. qingshengii CGMCC 1.12097T
S. sp. FXJ1.4098
M. muleiense CGMCC 1.11022T
S. coelicolor M145
Sinorhizobium meliloti 2011
S. sparsogenes 4.1446T
Sinorhizobium meliloti 2011
S. albidoflavus J1074
B
M. amorphae CGMCC 1.2541T
M. huakuii CGMCC 1.2543T
M. muleiense CGMCC 1.11022T
M. qingshengii CGMCC 1.12097T
M. sp. BAC0074
M. sp. BAC0120
Radius (mm)
Inhibition
n = 3
S. sp. FXJ1.264
S. sparsogenes 4.1446T
S. coelicolor M145
S. albidoflavus J1074
S. albidoflavus CR10
S. albidoflavus Ma1
S. sp. FXJ1.4034
S. sp. FXJ1.172
S. sp. FXJ1.4033
C
M. amorphae CGMCC 1.2541T
M. huakuii CGMCC 1.2543T
M. muleiense CGMCC 1.11022T
M. qingshengii CGMCC 1.12097T
M. sp. BAC0120
M. sp. BAC0074
Radius (mm)
Inhibition
Recovery
n = 3
S. sp. FXJ1.4098
S. sp. FXJ1.4034
S. sp. FXJ1.172
S. sp. FXJ1.4033
S. sp. FXJ1.4038

Fig. S18. Characterization of different pairwise interaction types between streptomycetes and rhizobia.

A. Representative pictures for three interaction types: competition (*Streptomyces* inhibited the growth of rhizobia), PBDM (*Streptomyces* inhibited the growth of rhizobia at a certain distance while not affecting those in the vicinity), and neutrality (both *Streptomyces* and rhizobia grew as well as their monocultures). Rhizobia were inoculated onto the culture plates four days after the inoculation of *Streptomyces* strains, and pictures were taken after four days of co-culture. A total of 77 pairs of interactions ($n = 3$) were tested, among which 38 pairs showed competition, 27 pairs showed PBDM, and 12 pairs showed neutrality. Representative pictures are shown for three pairs of each interaction type. **B.** Quantitative and statistical analyses of the radius of inhibition zone in competitive interactions. The radius of inhibition zone by *Streptomyces* ranged from 15.17 ± 0.55 to 39.97 ± 0.76 mm (mean ± standard deviation), and no recovery zone was detected in these interactions. **C.** Quantitative and statistical analyses of the radii of inhibition and recovery zones in PBDM interactions. The radius of inhibition zone by *Streptomyces* ranged from 20.33 ± 0.25 to 40.37 ± 0.40 mm (mean ± standard deviation) and the radius of recovery zone by *Streptomyces* ranged from 10.77 ± 1.45 to 14.20 ± 2.05 mm (mean ± standard deviation). Grey shadows (error bars) show the standard deviation from three biologically independent experiments.

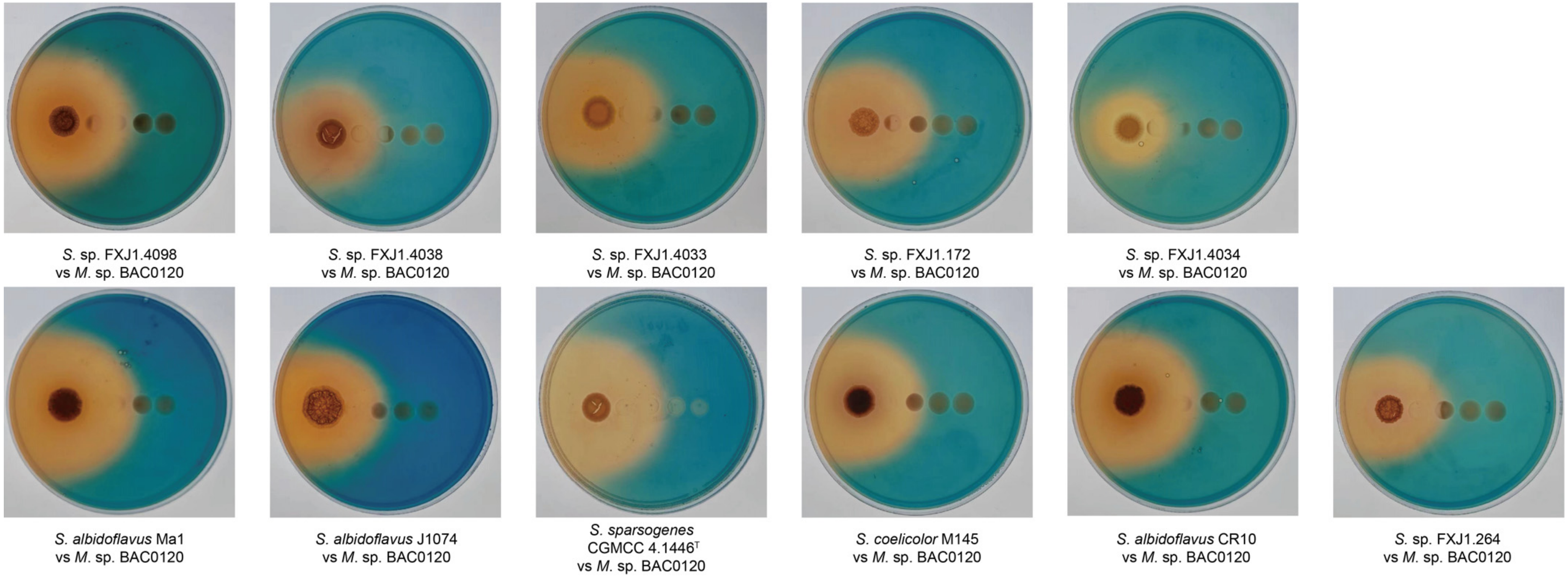

Fig. S19. Detection of siderophore distribution in the pairwise interaction plates of 11 *Streptomyces* strains and *M*. sp. BAC0120 by an overlay of chrome azurol S (CAS) agar (*n* = 3).

Orange areas indicate that iron is chelated by siderophores.

Supplementary Tables

Table S1. Strains used in this study

Name	Description	Habitat (origin)	Source and reference
Escherichia coli			
Top 10	For plasmid propagation	–	Invitrogen
***Streptomyces* strains**			
S. sp. FXJ1.4098	Wild-type, for PBM test	Red soil (Jiang Xi Province, China)	[2]
FXJ1.4098Δ*desD*	*desD* disruption mutant of *S.* sp. FXJ1.4098, for PBDM test	–	[2]
S. sp. FXJ1.4033	Wild-type, for PBDM test	Red soil (Jiang Xi Province, China)	[2]
S. sp. FXJ1.4034	Wild-type, for PBDM test	Red soil (Jiang Xi Province, China)	[2]
S. sp. FXJ1.4038	Wild-type, for PBDM test	Red soil (Jiang Xi Province, China)	[2]
S. sp. FXJ1.172	Wild-type, for PBDM test	Red soil (Jiang Xi Province, China)	[3]
S. sp. FXJ1.264	Wild-type, for PBDM test	Red soil (Jiang Xi Province, China)	[3]
S. albidoflavus J1074	Wild-type, for PBDM test	Soil (–)	[4]
S. albidoflavus Ma1	Wild-type, for PBDM test	Imperial moth (Guanacaste Conservation Area, Costa Rica)	[5]
S. albidoflavus CR10	Wild-type, for PBDM test	Imperial moth (Guanacaste Conservation Area, Costa Rica)	[5]

S. coelicolor M145	Wild-type, for PBDM test	–	[6]
S. sparsogenes CGMCC 4.1446^T	Wild-type, for PBDM test	Soil (–)	CGMCC
***Mesorhizobium* strains**			
M. sp. BAC0120	Wild-type, for PBDM test	Red soil (Jiang Xi Province, China)	[2]
ΔTBDR	TonB-dependent hemin receptor gene disruption mutant, for PBDM test	–	This study
Δ*hmuU*	*hmuU* disruption mutant, for PBDM test	–	This study
Δ*hmuV*	*hmuV* disruption mutant, for PBDM test	–	This study
M. sp. BAC0074	Wild-type, for PBDM point-to point-test	Red soil (Jiang Xi Province, China)	[2]
M. amorphae CGMCC 1.2541^T	Wild-type, for PBDM test	Root nodules of *Amorpha fruticosa* (Beijing, China)	CGMCC
M. huakuii CGMCC 1.2543^T	Wild-type, for PBDM test	Nodules of *Astragalus sinicus* (Nanjing, China)	CGMCC
M. muleiense CGMCC 1.11022^T	Wild-type, for PBDM test	Root nodules of *Cicer arietinum* (Shuangdamen villages, Qitai county, Xinjiang Province, China)	CGMCC
M. qingshengii CGMCC 1.12097^T	Wild-type, for PBDM test	Nodules of *Astragalus sinicus* (Jiang Xi Province, China)	CGMCC
***Sinorhizobium* strain**			
Sinorhizobium meliloti 2011	Wild-type, for PBDM test	–	-
**Plant pathogens**			
Agrobacterium rubi CGMCC 1.2555^T	Wild-type, for pairwise and tripartite interactions	–	CGMCC

Fusarium oxysporum f. sp. cucumerinum CGMCC 3.2830	Wild-type, for pairwise and tripartite interactions	–	CGMCC
Bipolaris sorokiniana ZB8	Wild-type, for pairwise and tripartite interactions	–	CGMCC
**Co-isolated bacteria**			
Bacillus sp. BAC0111	Wild-type, for pairwise and tripartite interactions	Red soil (Jiang Xi Province, China)	[2]
Massilia sp. BAC0116	Wild-type, for pairwise and tripartite interactions	Red soil (Jiang Xi Province, China)	[2]
Massilia sp. BAC0118	Wild-type, for pairwise and tripartite interactions	Red soil (Jiang Xi Province, China)	[2]
Microvirga sp. BAC0121	Wild-type, for pairwise and tripartite interactions	Red soil (Jiang Xi Province, China)	[2]
Noviherbaspirillum sp. BAC0123	Wild-type, for pairwise and tripartite interactions	Red soil (Jiang Xi Province, China)	[2]
Arthrobacter sp. FXJ1.4092	Wild-type, for pairwise and tripartite interactions	Red soil (Jiang Xi Province, China)	[2]
Arthrobacter sp. FXJ1.4093	Wild-type, for pairwise and tripartite interactions	Red soil (Jiang Xi Province, China)	[2]
Arthrobacter sp. FXJ1.4094	Wild-type, for pairwise and tripartite interactions	Red soil (Jiang Xi Province, China)	[2]
Micromonospora sp. FXJ1.4095	Wild-type, for pairwise and tripartite interactions	Red soil (Jiang Xi Province, China)	[2]
Streptomyces sp. FXJ1.4099	Wild-type, for pairwise and tripartite interactions	Red soil (Jiang Xi Province, China)	[2]
Streptomyces sp. FXJ1.4100	Wild-type, for pairwise and tripartite interactions	Red soil (Jiang Xi Province, China)	[2]

–, details of isolation unavailable.

CGMCC, China General Microbiological Culture Collection Center.

Table S2. Media used in this study

Medium	Composition
GYM	Yeast extract 4.0 g, malt extract 10.0 g, glucose 4.0 g, $CaCO_3$ 2.0 g, distilled water 1000 mL, agar 15 g if needed.
TY	Tryptone 5.0 g, yeast extract 3.0 g, $CaCl_2$ 0.6 g, distilled water 1000 mL, agar 15g if needed.
LB	Tryptone 10.0 g, yeast extract 5.0 g, NaCl 10.0 g, distilled water 1000 mL, agar 15g if needed.
PDA	Potato infusion made from 200.0 g potatoes, glucose 20.0 g, distilled water added to 1000 mL, agar 15 g if needed.
CAS	Chrome azurol S (CAS) agar [7].

Table S3. Plasmids used in this study

Name	Description	Source and reference
pJQ200SK	Suicide vector; P15A*sacB*, Gm^{r}	[8]
pJQ::*TBDR*	Used for disruption of TonB-dependent hemin receptor gene	This study
pJQ::*hmuU*	Used for disruption of *hmuU*	This study
pJQ::*hmuV*	Used for disruption of *hmuV*	This study
pRK2013	Helper plasmid for conjugation of pJQ200SK, kan^{r}	[9]

Table S4. Primers used in this study

**Name**	**Sequence (5'-3')**	**Purpose**
**For gene disruption**		
BAC-*hmuV*-F	ATATCGAATTCCTGCAGCCCGGCGACTGTCCTGTCCTTCC	*hmuV* disruption
BAC-*hmuV*-R	CTAGAACTAGTGGATCCCCCGCAATCAGCTCGTCCGACAG	
BAC-*hmuV*-tF	CAAATCCACGCTGCTCAAGAC	*hmuV* disruption verification
BAC-*hmuV*-tR	CGTGCCGACTTTCATGTGGC	
BAC-*hmuU*-F	ATATCGAATTCCTGCAGCCCTGCCGTGATGCAGGGACTGT	*hmuU* disruption
BAC-*hmuU*-R	CTAGAACTAGTGGATCCCCCAGGGCGTTACGGCAAGGGA	
BAC-*hmuU-t*F	GACAGAACGCCGACCTATCCG	*hmuU* disruption verification
BAC-*hmuU-t*R	TCGCCGTATGTTTCAGGCTCTG	
BAC-TBDR-F	ATATCGAATTCCTGCAGCCCAGCCATGTCGATCAGGAGCA	TBDR gene disruption
BAC-TBDR-R	CTAGAACTAGTGGATCCCCCGCCGCCCTTATAGTCGTCATAG	
BAC-TBDR-tF	AAAGCAGGATCAGGCTGAGAAC	TBDR gene disruption verification
BAC-TBDR-tR	GTCCGTATCATCGTCACCTTTCA	
**For RT-qPCR analyses**		
qBAC-16S-F	CCTTTGATACTGGCATTCTCG	For RT-qPCR analyses in heme uptake cluster
qBAC-16S-R	CTTCGCCACTGGTGTTCCT	
qBAC-*hmuV*-F	AAGGCGAAATCCTCTTCAATACCCG	
qBAC-*hmuV*-R	CACCGTGAAAGGGAAGGACAGGA	
qBAC-*hmuU*-F	CCCTTGCCGTAACGCCCTTTCT	
qBAC-*hmuU*-R	AGATCGCCGTATGTTTCAGGCTCTG	
qBAC-*hmuT*-F	TTGTCGGCGTTGCAGAATAAGTG	
qBAC-*hmuT*-R	AGCACTGACAAACGCTTGAACGAC	
qBAC-*hmuS*-F	GGCAGTGAAGAGCATAGGCGAGGAC	
qBAC-*hmuS*-R	CGACGAAGCACATGATCGGCAC	
qBAC-TBDR-F	CTGGCGTCGCCTTTCCCTTCCT	
qBAC-TBDR-R	AGCCTTCAGCCGCAACGCATC	
qFXJ-16S-F	GCTTGACATACATCGGAAACA	For RT-qPCR analyses in heme biosynthetic cluster
qFXJ-16S-R	CGCTCGTTGCGGGACTTA	
qFXJ-*tRNAglu*-F	ATGGAGATCACCCATGTGCTGC	

qFXJ-*tRNAglu*-R	CGCCCATCACATACGGCAGAT	
qFXJ-*hemA*-F	TCAGTCGCTCGTCACCTTCG	
qFXJ-*hemA*-R	GCCCGATCCAATGTCCTGTT	
qFXJ-*hemL*-F	TTCGTCTCCGAGTTCATCCAG	
qFXJ-*hemL*-R	TTGGTGGTGGTCTCGTAGGC	
qFXJ-*hemB*-F	CCTCGCCTACGCCGTCAAGT	
qFXJ-*hemB*-R	GCGGGCTTGACCATCACCA	
qFXJ-*hemC*-F	CTCGACGAGGCGACCGAGAT	
qFXJ-*hemC*-R	CGGGTGTACGGGTCGTCGAG	
qFXJ-*hemD*-F	TGCGGGTGGACGTACTGTCG	
qFXJ-*hemD*-R	CGTGCCCTCCTTCGTGAACC	
qFXJ-*hemE*-F	GGACCCGGACGATGTGAAGTAC	
qFXJ-*hemE*-R	CCTTGGTGCGCTCGTGGTT	
qFXJ-*hemY*-F	GCCACCTTCTCCAGCCGCAAAT	
qFXJ-*hemY*-R	GGCGAGCGACAGCCTTACCA	
qFXJ-*hemH*-F	CCTTCACCACGCACTCCATCC	
qFxJ-*hemH*-R	TGGTCGCAGATGTCCGGCTC	
qFXJ-*hemQ*-F	TCGCCGCCAAGGACGTCAC	
qFXJ-*hemQ*-R	TGGTGCGGCGGAAGAGGTT	

Primers underlined indicate overlapping sequences with the pJQ200SK plasmid for Gibson assembly of linear DNA fragments.

Table S5. Gene sets enriched in co-cultured *S.* sp. FXJ1.4098 at T1 compared to its monoculture

Gene set	Size	NES	*p* val	FDR q-val
Porphyrin and chlorophyll metabolism	40	1.452	0.000	0.216
Biosynthesis of amino acid	145	1.396	0.000	0.213
Sulfur metabolism	16	1.388	0.000	0.204
Arginine biosynthesis	23	1.365	0.000	0.220
Cysteine and methionine metabolism	34	1.349	0.000	0.211

Table S6. Relative expression levels of genes involved in heme biosynthesis in co-cultured and monocultured *S.* sp. FXJ1.4098 in transcriptome data

		T1			T2		
Gene	Gene ID	Monoculture group fpkm	Co-culture group fpkm	FC (co-culture vs monoculture)	Monoculture group fpkm	Co-culture group fpkm	FC (co-culture vs monoculture)
tRNAglu	peg.5792	7.108	10.414	1.465	6.283	8.186	1.303
hemA	peg.4158	11.535	15.235	1.321	11.971	11.947	0.998
hemL	peg.376	9.827	7.905	0.804	10.177	12.539	1.232
hemB	peg.12428	23.013	30.892	1.342	29.962	22.293	0.744
hemC	peg.3248	325.779	348.897	1.071	266.686	303.660	1.139
hemD	peg.5789	238.580	266.296	1.116	406.005	459.211	1.131
hemE	peg.3610	301.257	364.050	1.208	343.265	271.541	0.791
hemY	peg.3602	34.752	46.464	1.337	49.572	87.015	1.755
hemH	peg.3603	41.922	55.667	1.328	41.990	52.823	1.258
hemQ	peg.3601	142.151	131.243	0.923	118.799	126.094	1.061

Numbers in red highlight the up-regulated expression of the corresponding genes in the transcriptome analysis of co-cultured *S.* sp. FXJ1.4098 compared to the monoculture. FC, fold change. Fpkm, fragments per kilobase of transcript per million mapped reads.

References

1. Toriya M, Yaginuma S, Murofushi S, Ogawa K, Muto N, Hayashi M, et al. Zincphyrin, a novel coproporphyrin III with zinc from *Streptomyces* sp. J Antibiot (Tokyo). 1993;46:196–200.
2. Yan B, Liu N, Liu M, Du X, Shang F, Huang Y. Soil actinobacteria tend to have neutral interactions with other co-occurring microorganisms, especially under oligotrophic conditions. Environ Microbiol. 2021;23:4126–40.
3. Guo X, Liu N, Li X, Ding Y, Shang F, Gao Y, et al. Red soils harbor diverse culturable actinomycetes that are promising sources of novel secondary metabolites. Appl Environ Microbiol. 2015;81:3086–103.
4. Zaburannyi N, Rabyk M, Ostash B, Fedorenko V, Luzhetskyy A. Insights into naturally minimised *Streptomyces albus* J1074 genome. BMC Genomics. 2014;15:97.
5. Cheng K, Rong X, Pinto-Tomás AA, Fernández-Villalobos M, Murillo-Cruz C, Huang Y. Population genetic analysis of *Streptomyces albidoflavus* reveals habitat barriers to homologous recombination in the diversification of streptomycetes. Appl Environ Microbiol. 2015;81:966–75.
6. Kieser T, Bibb MJ, Buttner MJ, Chater KF, Hopwood DA. Practical *Streptomyces* genetics: John Innes Foundation Norwich; 2000.
7. Milagres AMF, Machuca A, Napoleão D. Detection of siderophore production from several fungi and bacteria by a modification of chrome azurol S (CAS) agar plate assay. J Microbiol Meth. 1999;37:1–6.
8. Quandt J, Hynes MF. Versatile suicide vectors which allow direct selection for gene replacement in gram-negative bacteria. Gene. 1993;127:15–21.
9. Ditta G, Stanfield S, Corbin D, Helinski DR. Broad host range DNA cloning system for gram-negative bacteria: construction of a gene bank of *Rhizobium meliloti*. Proc Natl Acad Sci USA. 1980;77:7347–51.
